# Genome-wide identification of genes encoding cystathionine beta synthase domain-containing proteins in wheat and its relationship with anther male sterility under heat stress

**DOI:** 10.3389/fpls.2022.1061472

**Published:** 2022-12-14

**Authors:** Hongzhan Liu, Qi Wang, Liuyong Xie, Kedong Xu, Fuli Zhang, Xianle Ruan, Lili Li, Guangxuan Tan

**Affiliations:** ^1^ College of Life Science and Agronomy, Zhoukou Normal University, Zhoukou, Henan, China; ^2^ School of Network Engineering, Zhoukou Normal University, Zhoukou, Henan, China; ^3^ Key Laboratory of Plant Genetics and Molecular Breeding, Zhoukou Normal University, Zhoukou, Henan, China; ^4^ Institute of Plant Protection and Edible Mushrooms, Zhoukou Academy of Agricultural Sciences, Zhoukou, Henan, China

**Keywords:** *Triticum aestivum* L., gene repetition events, cystathionine beta synthase, synteny analysis, gene expression

## Abstract

Cystathionine beta synthase (CBS) domains containing proteins (CDCPs) plays an important role in plant development through regulation of the thioredoxin system, as well as its ability to respond to biotic and abiotic stress conditions. Despite this, no systematic study has examined the wheat CBS gene family and its relation to high temperature-induced male sterility. In this study, 66 CBS family members were identified in the wheat genome, and their gene or protein sequences were used for subsequent analysis. The *TaCBS* gene family was found to be unevenly distributed on 21 chromosomes, and they were classified into four subgroups according to their gene structure and phylogeny. The results of collinearity analysis showed that there were 25 shared orthologous genes between wheat, rice and *Brachypodium distachyon*, and one shared orthologous gene between wheat, millet and barley. The cis-regulatory elements of the *TaCBS* were related to JA, IAA, MYB, etc. GO and KEGG pathway analysis identified these *TaCBS* genes to be associated with pollination, reproduction, and signaling and cellular processes, respectively. A heatmap of wheat plants based on transcriptome data showed that *TaCBS* genes were expressed to a higher extent in spikelets relative to other tissues. In addition, 29 putative tae-miRNAs were identified, targeting 41 *TaCBS* genes. Moreover, qRT-PCR validation of six *TaCBS* genes indicated their critical role in anther development, as five of them were expressed at lower levels in heat-stressed male sterile anthers than in Normal anthers. Together with anther phenotypes, paraffin sections, starch potassium iodide staining, and qRT-PCR data, we hypothesized that the genes encoding CDCPs has a very important connection with the heat-stressed sterility process in wheat, and these data provide a basis for further insight into their relationship.

## Introduction

The cystathionine beta synthase (CBS) domain, consisting of 60 amino acids, is a class of proteins found in almost all species, from archaea, bacteria, plants, to humans. The CBS structural domain, a group of conserved structural domains of proteins, was first discovered by Bateman in the genome of the archaeon methanococcus (Bateman, 1997). These domains are not limited to CBS but are also associated with several other functionally unrelated proteins, such as chloride channels (CLC), AMP-activated protein kinase (AMPK), and inosine-5’-mono-phosphate dehydrogenase (IMPDH) (Baykov et al., 2011). The importance of the CBS domain was gradually recognized through the discovery that point mutations in the CBS structural domains of human enzymes and membrane channels can cause several genetic diseases in humans (Baykov et al., 2011). Mutations in cystathionine beta synthase, an s-adenosylmethionine-regulating enzyme, lead to a metabolic disease called homocystinuria in humans (Shan et al., 2001). In addition, CBS domain-containing proteins (CDCPs) have also been identified in plants, with 34, 71, 59, and 106 CDCPs identified in *Arabidopsis*, soybean, rice, and cotton, respectively (Kushwaha et al., 2009; Hao et al., 2016; Ali et al., 2021). Furthermore, it has been shown that CDCPs are involved in plant development under salt stress, oxidative stress, drought stress, and extreme temperature treatments, playing an especially important role in biotic and abiotic stress tolerance (Kumari et al., 2009; Sahu and Shaw, 2009; Li et al., 2018; Kumar et al., 2018).

The plant CP12 protein forms a complex with the well-known redox-regulated Calvin cycle enzymes, namely glyceraldehyde-3-phosphate dehydrogenase (GADPH) and phosphate hydrogenase (PRK) (Michelet et al., 2013), and a fusion protein containing CP12 protein and CBS structural domain protein was found in cyanobacteria, suggesting that CBS structural domain protein may play a regulatory role in the redox-regulated system (Stanley et al., 2013; López-Calcagno et al., 2014). Additionally, studies in Arabidopsis have demonstrated that proteins consisting of a single CBS structural domain pair can regulate the thioredoxin system for the purpose of stabilizing cellular redox homeostasis and regulating plant development. CBSX1 and CBSX2, which are localized in chloroplasts, activate all the thioredoxin proteins in the thioredoxin-Trx system, including Trx f, Trx m, Trx x, and Trx y. CBSX1 in Arabidopsis is expressed in cotyledon and floral tissues, especially in anthers; CBSX3 is localized to the mitochondria and regulated mitochondrial Trx members in the NADP-Trx system; CBSX4 is localized in the cytosol, and CBSX5 and CBSX6 are localized in the endoplasmic reticulum, and they may have similar functions to CBSX1-3 in these subcellular organelles in *Arabidopsis* (Yoo et al., 2011). It was demonstrated that OsCBSX3 in rice is exclusively localized to the plasma membrane, and qRT-PCR results showed that significant up-regulation of OsCBSX3 transcripts resulted from either inoculation with magnaporthe oryzae or exogenous application of salicylic acid (SA) or methyl jasmonate (MeJA) (Mou et al., 2015). Furthermore, as a single protein containing the CBS structural domain in rice, CBSX4 exhibits antioxidant, salt and heavy metal resistance when overexpressed in the model plant tobacco. OsCBSCBSPB4, a protein containing two cysteine-β-synthase structural domains, was shown to play an important role in abiotic stress tolerance such as salt stress, drought stress, and high temperature stress (Kumar et al., 2018). Phenotypic analysis of *GmCBSDUF3* transgenic *Arabidopsis thaliana* reported enhanced tolerance to drought and salt stress in *Arabidopsis* plants overexpressing this soybean gene. The *GmCBS21* gene, which encodes a protein containing CBS structural domain, plays a role in the low nitrogen stress response in soybean (Hao et al., 2016). In perilla, the mature CbCBS protein directly or indirectly modulates cellular energy levels, thereby increasing mitochondrial ATP content during metabolic stress in senescent leaves (Zhu et al., 2007).

Reactive oxygen species within the anther determine the level of lignification within the secondary wall of the inner anther, all of which are associated with anther dehiscence (Bonner and Dickinson, 1989; Kawasaki et al., 2006). In *Arabidopsis*, it was found that CBSX1 and CBSX2 plays an important role in anther dehiscence. Their results show that signal transduction between hydrogen peroxide and jasmonic acid enhances the thickening and proliferation of secondary cell walls of the anther endothelial cells. It is also a key regulator of flowering (Jung et al., 2013). Furthermore, there is confirmation that knockout CBSX3 plants exhibit male sterility, which is mainly caused by anther indehiscence due to failure of secondary wall thickening of the inner anther wall (Shin et al., 2020). In addition, there has been evidence that miRNAs may play a role in plant temperature stress responses, pollen development, and male sterility. In cotton, a total of 382 miRNAs and 347 target genes were identified from the anthers of high-temperature-insensitive and high-temperature-sensitive cultivars, and miR156 was found to be inhibited by high temperature stress, activating the auxin signalling pathway (Ding et al., 2020). Treatment of cotton with exogenous IAA under heat stress leads to male sterility, implying that miR156 plays an important role in regulating crop fertility (Min et al., 2014). Furthermore, in wheat binuclear stage anthers, tae-miR1127b-3p was down-regulated under sterile conditions, while the target gene (*TapesCS3D01G464300*) associated with methyltransferase activity and methylation was up-regulated (Han et al., 2021).

The wheat (*Triticum aestivum* L.) genome is a stable heterohexaploid evolved from three subgroups A, B and D. Its genome is huge and complex, but it provides humans with proteins vitamins and minerals, and is a very important food crop worldwide (Consortium, T.I.W.G.S 2018; Liu et al., 2020; Hu et al., 2020). It is probably because of the allohexaploidy of the wheat genome that it is very difficult to exploit the heterosis of this important food crop. Although the utilization of heterosis in wheat is not as successful as that in maize rice, it is still one of the effective ways to greatly improve wheat yield. However, the basis of heterosis utilization is the selection and breeding of sterile wheat lines. In our recent study, we found that CBS domain-containing protein CBSX6-like, a hub differentially expressed gene in the transcriptome of Normal and male sterile anthers induced by high temperature of Zhoumai 28, showed significant down-regulation at the trinuclear stage of male sterile anthers, which may be related to the indehiscence of high-temperature sterile anthers leading to eventual sterility (Liu et al., 2021a). Moreover, an association between the generation of male sterility and CBS-related genes or proteins was found in both *Arabidopsis* and rice. In addition, research on the gene family encoding CDCPs has mainly concentrated on soybean, rice, cotton and *Arabidopsis* (Kushwaha et al., 2009; Ali et al., 2021; Hao et al., 2021), but there is no report on wheat. Therefore, considering the above studies, we speculate that genes encoding CDCPs may play an important role in regulating fertility, especially in indehiscence of sterile anther in wheat, and these issues are worthy of further exploration. In this study, the genome-wide members of the wheat gene family encoding CDCPs were identified by bioinformatics methods, and the physiological and biochemical properties, conserved motifs, cis-elements, gene collinearity and gene expression patterns of all family members were comprehensively analyzed. qRT-PCR was performed for the expression patterns of 6 *TaCBS* genes in wheat anther indehiscence under high-temperature stress conditions. These results provide a theoretical basis and technical reference for further analysis of the functional roles of the gene family encoding CDCPs in wheat male sterility.

## Materials and methods

### Plant materials, paraffin sectioning, and phenotype characteristics

Seeds of wheat (cv. Zhoumai 36) were dibble planted at the Experimental Field of Zhoukou Normal University in Zhoukou, Henan Province, People’s Republic of China (33°C64′ N, 114°C6 ′ E) on 21 October 2021. We divided the test plot into two plots with a row length of 100 cM, row width of 30 cM, and one plot with 10 rows. For one plot, a thin steel tube was used to support a piece of transparent plastic film (the highest height of the transparent plastic film was 1.5 m above the ground, effectively preventing the leaves from being burned). At the start of April, a light-transmitting plastic film was used to cover the wheat when it grew to the differentiation stage of pistil and stamen primordium (Feekes growth stage 8.5), and thus subjected to high-temperature stress for a week of continuous treatment at an average temperature of about 10 degrees above the outside temperature. The procedures were carried out as in a previous description (Liu et al., 2021a). There were 3 replicates for each anther sample. The anther samples were fixed by immersion in formalin–acetic acid–alcohol (FAA, formaldehyde 4ml, acetic acid 6ml, 50% ethanol 90ml) fixative and the air in the vial was pumped with a vacuum pump, so that the fixative could enter the anther tissue more completely. After 3 days, these anthers were transferred to 70% alcohol and stored at 4°C for subsequent paraffin sectioning. The longitudinal section slices were set to 12μm thickness and stained with periodic acid-Schiff (PAS) to detect insoluble carbohydrates (particularly polysaccharides and starch grains); the staining procedure was performed according to Song et al. (2015). KI-I_2_ staining technique was used to observe the starch accumulation in pollen grains, and staining results were used to preliminarily determine the fertility. Images were collected with an optical microscope.

### Identification and characteristics of the wheat CBS−domain−containing proteins

The Hidden Markov Model (HMM) profile (PF00571) corresponding to the Pfam CBS gene family was retrieved from Pfam database (Pfam 35.0: http://pfam.xfam.org/) to identify the CDCPs in wheat. Using the HMMER v3.0, we searched the sequence homologs from the wheat genome (IWGSC RefSeq v1.1: https://wheat-urgi.versailles.inra.fr/Seq-Repository/Assemblies), a high-quality protein collection (E-value < 1×10^-10^ and manual verification of an intact CDCPs) with which the predicted proteins obtained by CBS HMM were compared. Subsequently, a new wheat-specific CBS HMM was reconstructed using HMMER v3.0 kit hmmbuild and was used to select all proteins in wheat. Using the Compute pI/MW tool on the ExPasy website (http://au.expasy.org/tool.html), protein molecular weight and isoelectric point were predicted. The BUSCA web server (http://busca.biocomp.unibo.it/) was used to predict protein subcellular localization. A total of 121 candidate CDCPswere obtained from the wheat genome databases using the wheat-specific CBS HMM profile. After further verification by PFAM database and SMART database (Letunic and Bork, 2018; El-Gebali et al., 2018), 17 proteins were eliminated, and the remaining 104 proteins were further analyzed.

### Phylogenetic tree analysis of wheat gene family encoding CDCPs

The CBS protein sequences of *Arabidopsis*, rice, cocoa, and maize were retrieved and downloaded from the TAIR database (https://www.arabidopsis.org) and Ensembl Plants database (http://plants.ensembl.org/index.html). MEGA-X software was used to construct a phylogenetic tree of the gene family encoding CDCPs from the above plant species and wheat. The parameters were set as previously described (Liu et al., 2021b). Detailed parameters for phylogenetic tree construction: Algorithm of Neighbor-Joining, poission correction, and bootstrap repeated value 1000 times.

### Analysis of protein domain, exon and intron structure, and conserved motif of CDCPs encoded by gene family

GSDS software (http://gsds.cbi.pku.edu.cn/), MEME online tool (http://meme-suite.org/tools/meme) and NCBI-CDD website (https://www.ncbi.nlm.nih.gov/Structure/cdd/wrpsb.cgi) were used to analysis gene structure, motifs (the number of different motifs, 20; minimum width of motifs, six; maximum width of motifs, 200);, and domain distribution, respectively. Finally, these files were optimized using TBtools (Chen et al., 2020).

### Cis−element prediction of gene family encoding CDCPs

Based on the chromosome location information of the wheat genome, the biolinux system was used to extract the 2.0kb promoter sequence upstream of the CDS of the CBS gene family member, and converted into the FASTA file format. Subsequently, these sequences were uploaded to PlantCare (http://bioinformatics.psb.ugent.be/webtools/plantcare/html/) for cis-element prediction. Furthermore, BioSequence viewer kit in TBtools was used to visualize these cis-elements.

### Chromosomal location and collinearity analysis among species

Blast alignment was performed on CBS gene family proteins sequence by Biolinux. The corresponding fragment duplications and tandem duplications of the CBS gene family were screened according to the comparison results. Chromosome location information and gene duplication results were optimized using the Gene Location Visualize kit and Advanced Circos kit in the TBtools software. All protein sequences and gff3 files of five species were downloaded from the Ensembl Plants database. Two-way comparison was performed by the blast tool in the TBtools software. The collinearity blocks (Minimum block size was set to 30) of all genes were obtained from MCscanX Wrapper. The collinearity comparison map of these five species was drawn by the Multiple Synteny Plot tool in the TBtools software (Chen et al., 2020).

### Transcriptomic data analysis of wheat gene family encoding CDCPs

To observe the *TaCBS* gene expression in different wheat tissues, the wheat RNA-Seq data were downloaded from WheatOmics 1.0 (http://wheatomics.sdau.edu.cn/) as depicted in a previous study (Liu et al., 2021b; Ma et al., 2021). TBtools software was used to perform log normalization on the downloaded TPM (Transcripts Per Kilobase Million) values, and to draw the expression heatmap of wheat genes encoding CDCPs.

### GO and KEGG pathway enrichment analysis

The query2go file and query2ko file were obtained by rapidly annotating the wheat species protein sequences with the EGGNOG-Mapper (http://eggnog-mapper.embl.de/). Gene ontology enrichment analysis and KEGG Pathway Enrichment were conducted and visualized with TBtools.

### Prediction of putative miRNAs targeting genes encoding CDCPs

Using the psRNATarget website (Dai et al., 2018; Raza et al., 2022), with parameters set to default, all *TaCBS* CDSs were used to predict miRNA target sites. The miRNA target network map for genes encoding CDCPs was generated by Cytoscape_v3.9.1 (Shannon et al., 2003).

### Extraction of total RNA, primer design, and quantitative real-time PCR analysis

All anther samples from each developmental stage were quick-frozen in liquid nitrogen after collection, followed by rapid transfer and storage in a -80°C freezer for subsequent qRT-PCR experiments. The total RNA of anthers was extracted by TRNzol Universal (Tiangen); cDNA was synthesized by first-strand cDNA synthesis kit (RevertAid Premium Reverse Transcriptase), and the above experiments were carried out according to the kit instructions. Primer premier 5.0 software was used to design the specific primers for qRT−PCR (Quantitative Real-Time PCR). The primer sequence details are provided in **Supplementary Table 1**. qRT−PCR were performed as previously described (Liu et al., 2021a). The wheat actin gene was used as an internal control and the expression level was calculated by the 2**^–⊿⊿Ct^** method.

## Results

### Identification, characterization and phylogenetic analysis of genes encoding CDCPs in wheat

As shown in **Table 1**, the CDS lengths ranged from 606bp to 2358bp; The length and molecular weight of these proteins ranged from 201 to 785 amino acids and 38.88kD to 83.62kD, respectively; The theoretical isoelectric points (pIs) of 82 proteins were acidic (4.82-6.89), and 22 proteins were alkaline (7.24-9.14). Considering that some of these protein sequences were translated from variable transcripts of one gene, we chose the longest transcript sequence as a representative and named it *TaCBS1*-*TaCBS66* according to the arrangement of chromosomal location (**Table 1**). Furthermore, the subcellular location information indicated that most of the CBS members were predicted to target the nucleus and chloroplast. A few CBS members were localized to the mitochondrion, cytoplasm and endomembrane system. These also indicate that this gene family may be functionally differentiated during evolution.

**Table 1:** Information about the TaCBS members in wheat.

Gene Name	Gene Locus	CDS Length (bp)	AA^a^	MW^b^ (kDa)	pI^c^	TMD^d^	SLP^e^
*TaCBS1*	TraesCS1A02G170000.1	1122	373	38.88	5.35	0	mitochondrion
*TaCBS2*	TraesCS1B02G187200.1	1122	373	38.95	5.47	0	mitochondrion
*TaCBS3*	TraesCS1D02G167600.1	1122	373	38.93	5.47	0	mitochondrion
*TaCBS4*	**TraesCS2A02G177000.1**	1362	453	48.06	4.82	0	cytoplasm
	TraesCS2A02G177000.2	1035	344	36.28	5.36	0	cytoplasm
*TaCBS6*	TraesCS2A02G281700.1	1278	425	46.95	5.45	0	nucleus
*TaCBS6*	TraesCS2A02G289200.1	1047	348	38.64	5.92	0	nucleus
	**TraesCS2A02G289200.2**	1371	456	50.92	5.56	0	nucleus
	TraesCS2A02G289200.3	1365	454	50.68	5.56	0	nucleus
	TraesCS2A02G289200.4	1314	437	48.82	5.58	0	nucleus
*TaCBS7*	TraesCS2A02G360600.1	1515	504	54.71	6.61	0	chloroplast
*TaCBS8*	TraesCS2B02G002100.1	666	221	23.38	7.87	0	chloroplast thylakoid membrane
*TaCBS9*	TraesCS2B02G203900.1	1260	419	44.39	4.97	0	cytoplasm
	**TraesCS2B02G203900.2**	1362	453	47.96	4.82	0	cytoplasm
	TraesCS2B02G203900.3	1149	382	40.74	5.00	0	nucleus
*TaCBS10*	TraesCS2B02G299000.1	1278	425	46.91	5.62	0	nucleus
*TaCBS11*	TraesCS2B02G305800.1	1050	349	38.69	6.00	0	nucleus
	TraesCS2B02G305800.2	1239	412	45.59	5.50	0	extracellular space
	TraesCS2B02G305800.3	1185	394	43.76	6.15	0	nucleus
	TraesCS2B02G305800.4	1371	456	50.89	5.74	0	nucleus
	TraesCS2B02G305800.5	1365	454	50.65	5.74	0	nucleus
	**TraesCS2B02G305800.6**	1395	464	51.83	5.74	0	nucleus
	TraesCS2B02G305800.7	1068	355	39.38	5.83	0	nucleus
	TraesCS2B02G305800.8	1164	387	43.34	6.76	0	nucleus
	TraesCS2B02G305800.9	1032	343	38.22	5.64	0	extracellular space
*TaCBS12*	TraesCS2D02G015500.1	666	221	23.52	8.73	0	chloroplast thylakoid membrane
	**TraesCS2D02G015500.2**	672	223	23.94	8.71	0	chloroplast thylakoid lumen
*TaCBS13*	**TraesCS2D02G185000.1**	1362	453	48.00	4.82	0	cytoplasm
	TraesCS2D02G185000.2	1035	344	36.33	5.23	0	cytoplasm
*TaCBS14*	TraesCS2D02G280600.1	1278	425	46.95	5.45	0	nucleus
*TaCBS15*	TraesCS2D02G287200.1	1047	348	38.70	5.65	0	nucleus
	TraesCS2D02G287200.2	1365	454	50.76	5.45	0	nucleus
	TraesCS2D02G287200.3	1068	355	39.48	5.42	0	nucleus
	**TraesCS2D02G287200.4**	1371	456	51.00	5.45	0	nucleus
	TraesCS2D02G287200.5	1164	387	43.45	6.51	0	nucleus
*TaCBS16*	TraesCS2D02G599900.1	1149	382	39.76	4.92	0	nucleus
*TaCBS17*	TraesCS3A02G226700.1	1206	401	44.09	5.67	0	nucleus
*TaCBS18*	TraesCS3A02G427100.1	1197	398	42.18	6.01	0	nucleus
*TaCBS19*	TraesCS3A02G429700.1	1290	429	46.99	5.14	0	nucleus
*TaCBS20*	TraesCS3A02G433400.1	1125	374	40.66	6.23	1	endomembrane system
	**TraesCS3A02G433400.2**	1653	550	58.85	7.25	1	nucleus
*TaCBS21*	TraesCS3A02G445200.1	1632	543	58.45	6.10	1	chloroplast
*TaCBS22*	TraesCS3B02G257800.1	1209	402	44.21	5.70	0	nucleus
*TaCBS23*	TraesCS3B02G463800.1	1197	398	42.21	5.93	0	nucleus
*TaCBS24*	TraesCS3B02G467600.1	1323	440	47.71	5.22	0	nucleus
*TaCBS25*	TraesCS3B02G469200.1	1125	374	40.62	6.22	1	endomembrane system
	**TraesCS3B02G469200.2**	1650	549	58.75	7.24	1	nucleus
*TaCBS26*	TraesCS3B02G479900.1	1392	463	49.86	5.02	1	chloroplast
*TaCBS27*	TraesCS3B02G573900.1	1128	375	40.53	5.42	0	nucleus
*TaCBS28*	TraesCS3D02G224700.1	1218	405	44.19	5.45	0	nucleus
*TaCBS29*	TraesCS3D02G422700.1	1197	398	42.23	5.93	0	nucleus
*TaCBS30*	TraesCS3D02G425000.1	1293	430	47.07	5.20	0	nucleus
*TaCBS31*	TraesCS3D02G426800.1	1656	551	59.03	7.25	1	nucleus
*TaCBS32*	TraesCS3D02G438100.1	1632	543	58.54	6.23	1	chloroplast
*TaCBS33*	**TraesCS3D02G513700.1**	1578	525	56.14	6.77	1	chloroplast
*TaCBS34*	**TraesCS4A02G206600.1**	1635	544	58.23	6.40	1	chloroplast
*TaCBS35*	TraesCS4A02G247300.1	618	205	22.40	9.14	0	mitochondrion
*TaCBS36*	TraesCS4A02G320000.1	1494	497	54.40	6.25	0	nucleus
	TraesCS4A02G320000.2	1467	488	53.55	6.34	0	nucleus
*TaCBS37*	TraesCS4B02G022400.1	1464	487	54.21	6.42	4	endomembrane system
*TaCBS38*	TraesCS4B02G067600.1	642	213	23.39	9.10	0	mitochondrion
*TaCBS39*	TraesCS4B02G110100.1	1632	543	58.02	6.25	1	chloroplast
	**TraesCS4B02G110100.2**	1764	587	63.71	5.81	0	nucleus
*TaCBS40*	TraesCS4D02G066600.1	618	205	22.40	9.14	0	mitochondrion
*TaCBS41*	TraesCS4D02G107800.1	1635	544	58.17	6.58	1	chloroplast
*TaCBS42*	TraesCS5A02G053000.1	618	205	23.05	7.92	0	mitochondrion
*TaCBS43*	TraesCS5A02G118800.1	1635	544	58.79	6.78	1	chloroplast
*TaCBS44*	TraesCS5A02G209500.1	1623	540	58.71	6.59	1	mitochondrion
*TaCBS45*	TraesCS5B02G063400.1	618	205	23.10	8.65	0	mitochondrion
*TaCBS46*	TraesCS5B02G117400.1	1464	487	52.77	6.30	0	chloroplast
	**TraesCS5B02G117400.2**	1641	546	59.02	7.70	1	chloroplast
*TaCBS47*	TraesCS5B02G207600.1	1626	541	58.75	6.40	1	mitochondrion
*TaCBS48*	**TraesCS5B02G559100.1**	1494	497	54.31	6.05	0	nucleus
	TraesCS5B02G559100.2	1467	488	53.49	6.14	0	nucleus
*TaCBS49*	TraesCS5D02G064300.1	618	205	23.05	7.92	0	mitochondrion
*TaCBS50*	**TraesCS5D02G130200.1**	1629	542	58.49	6.82	1	chloroplast
	TraesCS5D02G130200.2	1452	483	52.28	6.13	0	chloroplast
*TaCBS51*	TraesCS5D02G215700.1	1626	541	58.81	8.40	1	mitochondrion
*TaCBS52*	TraesCS5D02G565200.1	1125	374	41.04	6.60	0	extracellular space
	TraesCS5D02G565200.2	1467	488	53.59	6.31	0	nucleus
	**TraesCS5D02G565200.3**	1494	497	54.42	6.22	0	nucleus
*TaCBS53*	TraesCS6A02G132700.1	1317	438	46.55	5.17	0	nucleus
*TaCBS54*	TraesCS6A02G235600.1	1176	391	40.70	5.41	0	mitochondrion
*TaCBS55*	TraesCS6A02G283600.1	1791	596	63.61	6.06	7	endomembrane system
	**TraesCS6A02G283600.3**	2346	781	83.05	6.77	9	organelle membrane
*TaCBS56*	TraesCS6A02G392100.1	651	216	23.59	8.64	0	chloroplast
	**TraesCS6A02G392100.2**	654	217	23.72	8.64	0	chloroplast
	TraesCS6A02G392100.3	636	211	23.11	8.30	0	chloroplast
*TaCBS57*	TraesCS6B02G160900.1	1311	436	46.51	5.11	0	nucleus
*TaCBS58*	TraesCS6B02G264200.1	1167	388	40.53	5.35	0	nucleus
*TaCBS59*	TraesCS6B02G432300.1	654	217	23. 41	7.62	0	nucleus
	**TraesCS6B02G432300.2**	657	218	23.54	7.62	0	nucleus
	TraesCS6B02G432300.3	606	201	21.57	6.89	0	chloroplast
*TaCBS60*	TraesCS6D02G122400.1	1323	440	46.85	5.37	0	nucleus
*TaCBS61*	TraesCS6D02G218300.1	1158	385	40.07	5.43	0	nucleus
*TaCBS62*	TraesCS6D02G264100.1	2358	785	83.62	6.59	9	organelle membrane
*TaCBS63*	TraesCS6D02G378000.1	651	216	23.49	8.31	0	chloroplast
	TraesCS6D02G378000.2	636	211	23.01	7.64	0	chloroplast
	**TraesCS6D02G378000.3**	747	248	26.92	6.22	0	nucleus
	TraesCS6D02G378000.4	606	201	21.77	7.66	0	chloroplast
*TaCBS64*	TraesCS7A02G240700.2	2232	743	79.25	6.19	8	endomembrane system
*TaCBS65*	TraesCS7B02G136300.1	2232	743	79.11	6.19	8	endomembrane system
*TaCBS66*	TraesCS7D02G239700.2	2232	743	79.23	6.10	8	endomembrane system
	**TraesCS7D02G239700.3**	2241	746	79.55	6.49	8	endomembrane system

^a^Length of the amino acid sequence. ^b^Molecular weight of the amino acid sequence. ^c^Isoelectric point of the TaCBS proteins. ^d^Number of transmembrane domains, as predicted by the TMHMM server. ^e^Protein subcellular localization prediction by the BUSCA web server. The bold values indicate the selected transcript representing this gene. All TaCBS genes in the table indicate genes encoding cystathionine beta synthase domain-containing proteins.

**Figure 1:**
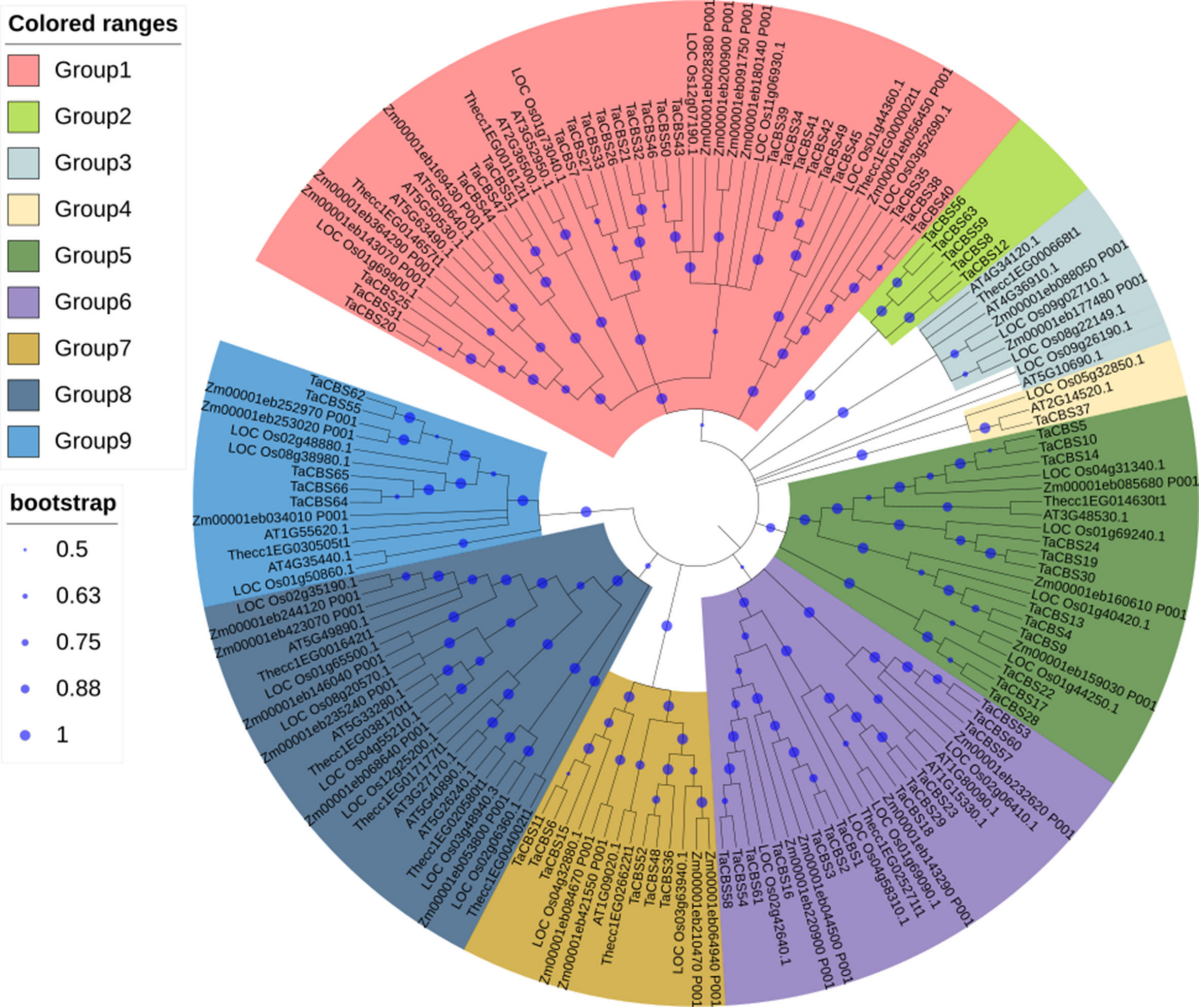
CBS proteins from five species characterized by their phylogenetic relationships and subfamilies. Phylogenetic tree constructed using MEGA X with 1000 bootstrap values shows relationships between CBS proteins from different plant species. There are different colors for the 9 subgroups, and the new names and accession numbers in wheat are listed in **Table 1**.

An unrooted phylogenic tree was constructed using MEGAX with the neighbor-joining (NJ) method according to the 159 CDCPs(All protein sequences are provided in **Supplementary Table 2**) corresponding to 66 CDCPs in wheat, the 30 CDCPs in rice, the 13 CDCPs in cacao, the 30 CDCPs in maize, and the 20 CDCPs in *Arabidopsis*. Among them, monocotyledons contain wheat, rice and maize; dicotyledons contain cocao and *Arabidopsis*. These proteins were divided into nine groups based on their sequence similarity, and the 66 TaCBSs were distributed into seven groups, with 24 members in group 1, 5 members in group 2, 1 member in group 4, 12 members in group 5, 13 members in group 6, 6 members in group7, and 5 members in group 9. The other two clades were clusters of *Arabidopsis*, maize, rice, and cacao. From the perspective of homology, *Arabidopsis* and cocoa, rice and maize often appear on the same branch in all subgroups, and they seem to be more closely related (**Figure 1**).

### Sequence features of gene structure, motifs and conserved domains of the TaCBS gene family

**Figure 2:**
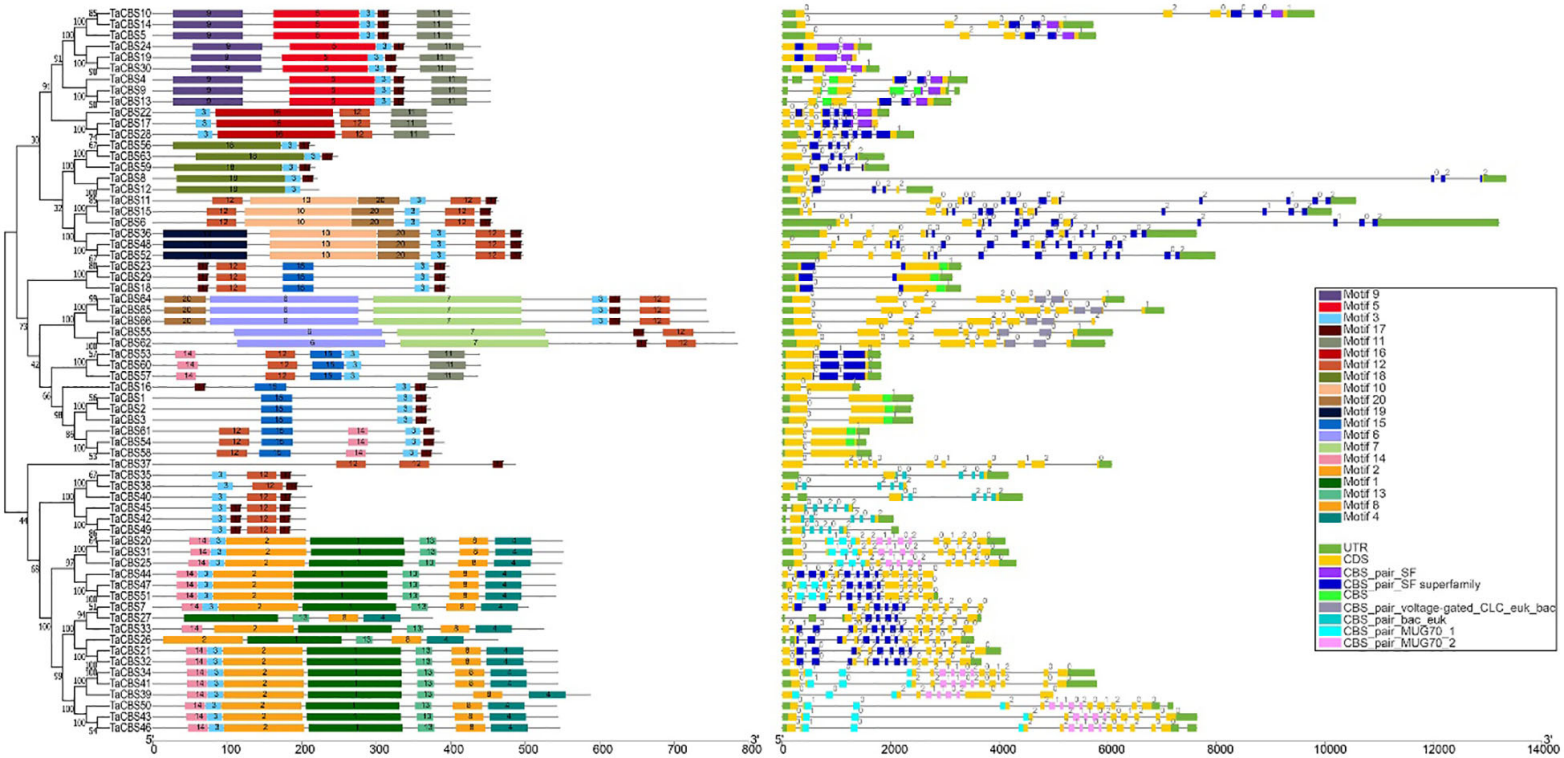
Phylogenetic relationship, conserved motifs, gene structure, and conserved domain of TaCBS proteins. Different colors are used to represent different motifs in the figure and in the upper right corner. Black lines represent non-conserved sequences in MEME results and introns in the exon-intron structure, respectively. The phylogenetic tree is constructed similarly to **Figure 1**.

In order to study their functional regions, the MEME online tool was used to predict the identified CDCPs sequences. The higher the homology of the protein sequence, the stronger the similarity of its motif arrangement. The phylogenetic tree of 66 CDCPs showed that these proteins can be divided into two major branches; one branch contains 41 proteins, and the other contains 25 proteins, and all proteins contain Motif 3 (**Figure 2**). Except for the shared motif, most of the first major branch contains motif 17 and motif 12. In addition, each different subgroup has its own unique motif, such as TaCBS10-13 (order on the phylogenetic tree) subgroup contains motif 5, 9 and 11. The second largest branch, TaCBS37 is a single branch, containing two motif 12 and one motif 17, and the remaining 24 proteins are divided into two subgroups, one containing motif 3, 12, 17; the other subgroup adds many new motifs, such as motif 1, 2, 4, 8, 13, 14 (**Figure 2**). Furthermore, gene structural diversity and conserved domains of the TaCBS gene family in wheat were investigated by studying the exon-intron organization and NCBI-CDD prediction. Although most of the genes at the fulcrum of the phylogenetic tree have similar exon architectures, similar CDS lengths have varied gene complete lengths, demonstrating that intron lengths are variable (e.g., *TaCBS10* and *TaCBS14*). The intron phase, or the position of the intron in the gene in relation to the three nucleotides of the genetic code, was studied. Intron phase 0 is described as an intron located between two full codes. Intron phase 1 and intron phase 2 are defined as the first intron and the next two nucleotides within the codon, respectively. Intron phase 0 is present in all 66 *TaCBS* members, with 8 being the most (such as *TaCBS11-TaCBS52* on the phylogenetic tree (**Figure 2**). Most genes are also distributed with intron phase 1 and intron phase 2, and some individuals only have intron phase 0 and 1 or only intron phase 0 and 2. In addition, on the upstream and downstream sides of 7 genes, there are no UTR regions, including *TaCBS7*, *17*, *19*, *33*, *39*, *48*, *56*. The protein’s CBS conserved domains were also predicted, and different types of CBS domains are colored differently in the gene structure diagram (**Figure 2**). In addition, for a more intuitive presentation and further verification of these conserved domains, we also used the SMART web site (http://smart.embl-heidelberg.de) to make predictions, and the results are presented in **Figure S1**. The prediction results are basically consistent with the NCBI-CDD prediction. According to the classification results, 66 TaCBS proteins were classified into 6 classes, namely CBSX1-25 (25 proteins), CBSCBS1-11 (11proteins), CBSCBSCBD1-6 (6 proteins), CBSCLC1-5 (5 proteins), CBSDUF1 (1protein) and CBSCBSPB1-1-18 (18 proteins). More details are available in **Figure S1** and **Supplementary Table 3**.

### Chromosome distribution and gene duplication of wheat TaCBS gene family

**Figure 3:**
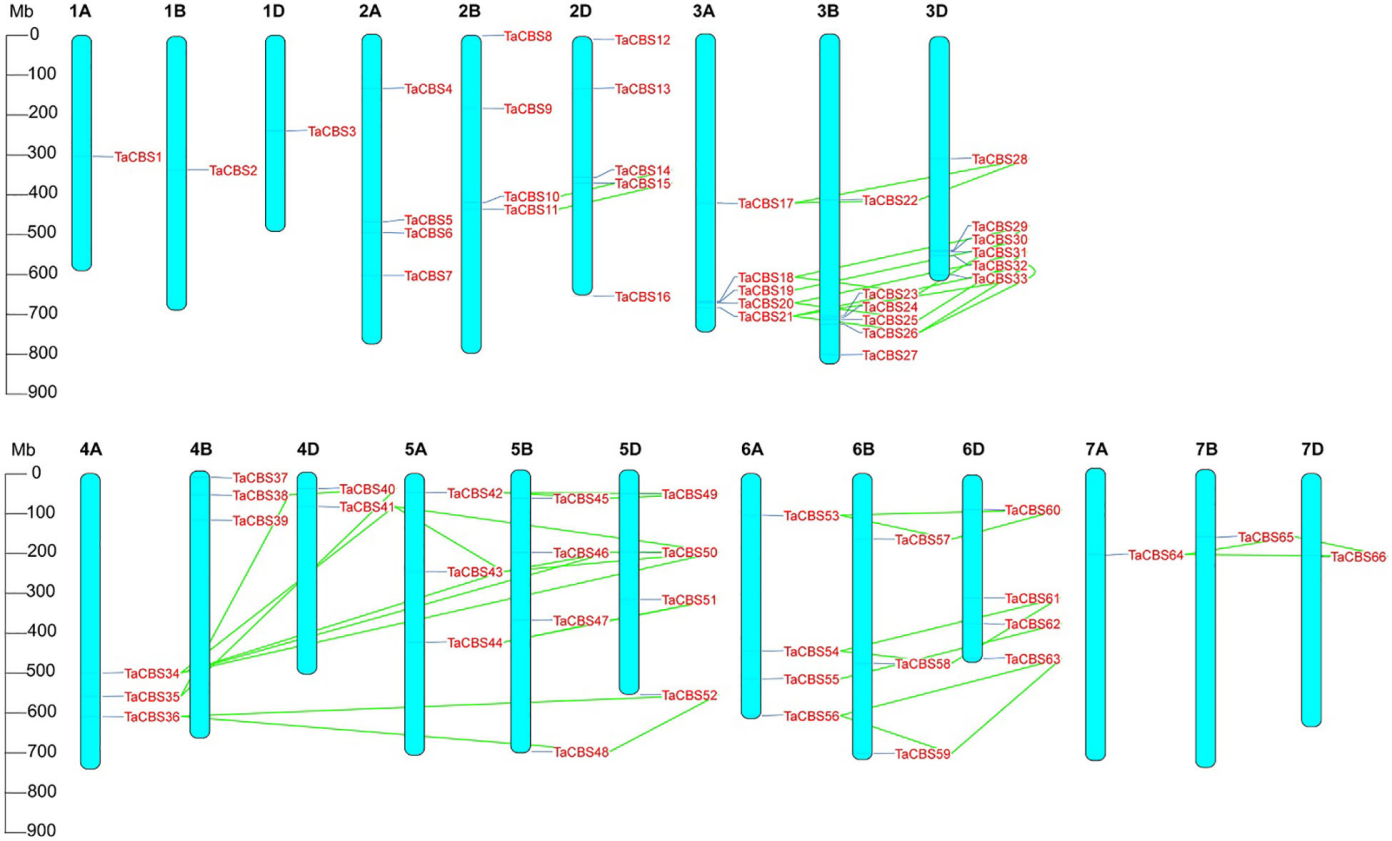
Chromosomal localization of the genes encoding CDCPs in the wheat genome. The positions on the linkage map have been determined. 66 TaCBS members are mapped to 21 chromosomes (1A-7A, 1B-7B, and 1D-7D). Green colored lines represent the connections between duplication events. Megabase pairs (Mb) are used to measure the scale.

The distribution and density of genes encoding CDCPs are uneven on 21 wheat chromosomes. Chromosomal localization of genes encoding CDCPs is displayed in **Figure 3**, which shows that most TaCBS members were tandemly distributed. There are 1 (in 1A, 1B, 1D, 7A, 7B, 7D), 2 (in 4D), 3 (in 4A, 4B, 5A, 6B), 4 (in 2A, 2B, 5B, 5D, 6A, 6D), 5 (in 2D, 3A) and 6 (in 3B, 3D) TaCBS members in different chromosomes, respectively. 78 percent (52/66) of wheat *TaCBS* members showed repetitive events. In **Figure 3**, the link regions of segment duplications on different chromosomes are connected by green colored lines. Fragment duplication produced multiple CBS homologs located on different chromosomes. Most fragment repetitive events occurred on the identically numbered homologous chromosomes, such as *TaCBS17* (3A), *TaCBS22* (3B) and *TaCBS28* (3D). Interestingly, fragment duplication events were also observed on partial homologous chromosomes with different numbers 4A, 4D and 5A, 5B, 5D, such as *TaCBS34* (4A), *TaCBS41* (4D) linked to *TaCBS43* (5A), *TaCBS46* (5B), *TaCBS50* (5D). Furthermore, we found that these genes with fragment replication events all clustered into the same branch of the evolutionary tree, e.g., evolutionary branches *TaCBS34*, *41*, *39*, *50*, *43*, *46*, and *TaCBS36*, *52*, *48*. These homologous genes have homologous sites on three or two partial homologous chromosomes, indicating that the wheat genes encoding CDCPs has a large number of homologous sites, showing a high homology retention rate. The conserved positions of these fragment replication regions located on different chromosomes suggests that fragment replication events play an important role in the expansion of the number of *TaCBS* members in wheat.

### Localization and synteny of the genes encoding CDCPs in the wheat genome

**Figure 4:**
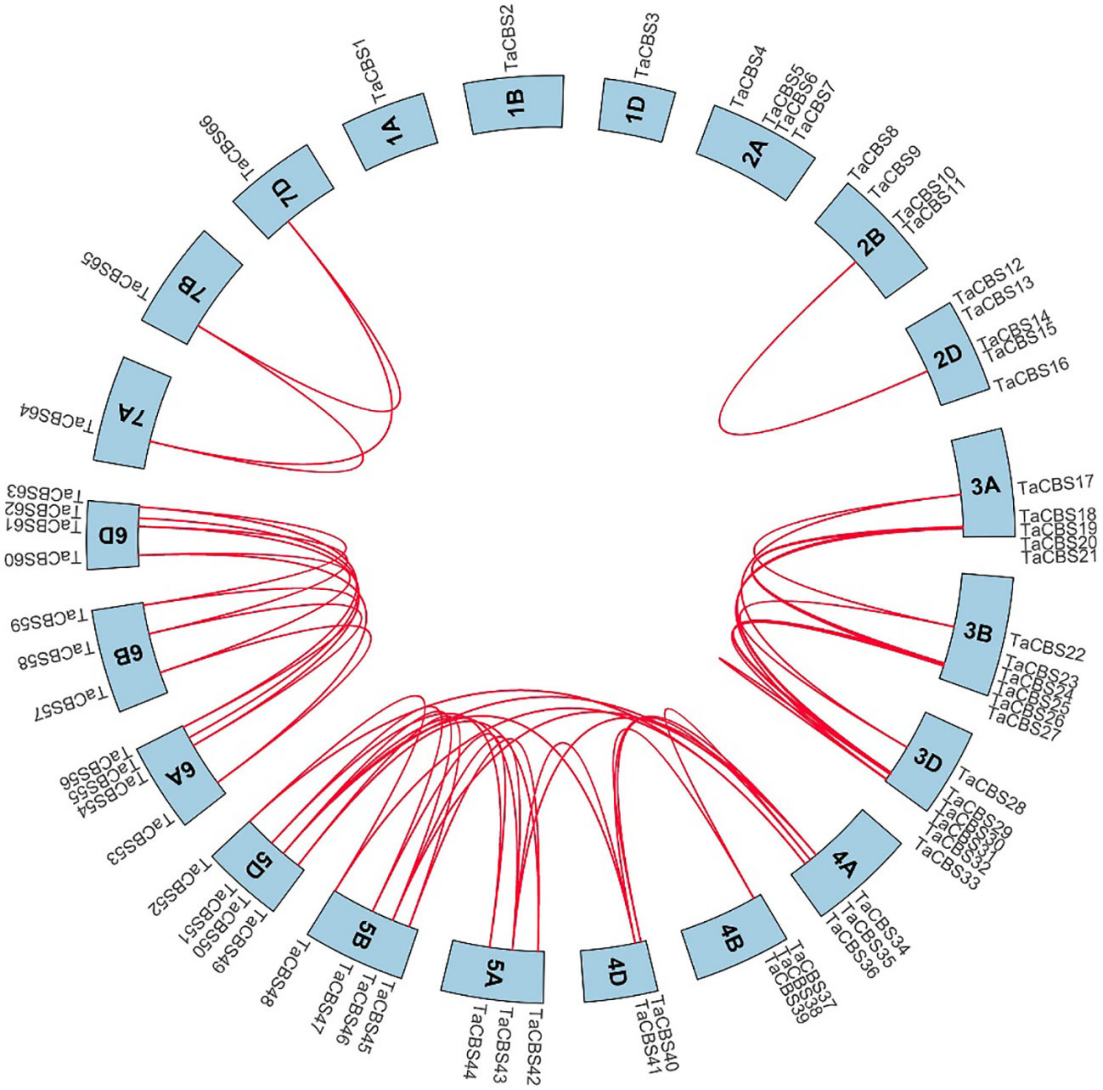
Genome-wide syntenic and localization analysis of the genes encoding CDCPs in wheat. The genes encoding CDCPs in wheat were mapped to different chromosomes with a cyan color. The red line indicates the synteny of gene pairs within the *TaCBS*. On the chromosome’s outermost side are the *TaCBS* members names.

We analyzed the collinearity of these genes encoding CDCPs in the wheat genome by using the Bio-linux system with the two-way blast comparison analysis and the MCScanX tool, and a total of 52 pairs of collinearity genes were identified. The Circos diagram shows the gene pairs with a syntenic relationship by a red line (**Figure 4**). Between 4A, 4D and 5A, 5B, 5D, there were cross-chromosomal paralogous homologous gene duplication events in addition to paralogous homologous genes of the same chromosome group. The detailed data is shown in **Supplementary Table 4**.

### Strong purifying selection for the *TaCBS* gene pairs in wheat

In genetics, Ka/Ks expresses the ratio between the rate of nonsynonymous mutations (Ka) and the rate of synonymous mutations (Ks) for two protein-coding genes. This ratio determines whether there is a selective pressure acting on this protein-coding gene. Ka/Ks with values equal to 1, less than 1, or greater than 1, indicates gene neutral evolution, purification selection, or positive selection, respectively. A further analysis of these replicated gene pairs revealed the Ka/Ks values of all wheat *TaCBS* members were less than 1, with a maximum value of 0.451. *TaCBS11*_*TaCBS15* constituted the corresponding gene pair (**Figure S2**; **Supplementary Table 5**).

### Synteny analysis of genes encoding CDCPs between wheat and four representative plant species

**Figure 5:**
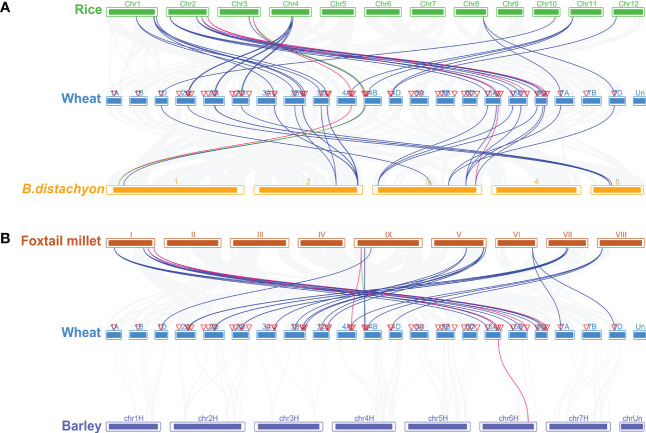
Homologous gene pairs between wheat and **(A)** Rice, and B.distachyon, **(B)** Foxtail millet, and Barley. There are gray lines in the background that indicate the collinear blocks within wheat genomes and other four plant genomes, while other colored lines highlight the collinear blocks centered on the A, B, and D chromosomes for wheat. It should be noted that the species names “Wheat”, “B.distachyon”, “Rice”, “Barley”, and “Foxtail millet” refer to *Triticum aestivum*, *Brachypodium distachyum*, *Oryza sativa*, *Hordeum vulgare*, and *Setaria italica*, respectively. Chromosomes are colored differently in different species.

To gain a deeper understanding of the phylogenetic mechanisms of *TaCBS* from wheat, we constructed four comparative syntenic maps with rice, *Brachypodium distachyum*, foxtail millet, and barley (**Figure 5**). In the syntenic map of wheat with rice and *Brachypodium distachyum*, we found that 37 genes in wheat were collinear with 18 genes in rice and 13 genes in *Brachypodium distachyum*. Among them, 25 genes have a collinear relationship with rice and *Brachypodium distachyum*, accounting for 68%. Ten of the remaining twelve genes are only collinear with rice, and the other two are only collinear with *Brachypodium distachyum* (*TaCBS20*, *TaCBS31*). In addition, the twenty-nine genes without collinearity were relatively conserved in wheat, neither with the *CBS* gene in rice nor with the *CBS* gene in *Brachypodium distachyum*. (**Figure 5A**; **Supplementary Table 6**). The results of collinearity analysis of wheat, foxtail millet (*Setaria italica*) and barley (*Hordeum vulgare* L.) revealed a total of 34 pairs of orthologous genes in wheat and foxtail millet, unevenly distributed on 16 loci on the 9 chromosomes, excluding chromosomes II, III, IV of foxtail millet, ranging from 1 to 3, respectively. Only one collinearity gene, *HORVU6HR1G071200.3*, is located on chr6H chromosome in barley, corresponding to the 6A chromosome of wheat (*TaCBS55*) (**Figure 5B**; **Supplementary Table 7**).

### Cis-element analysis of wheat CBS family gene promoter sequence

**Figure 6:**
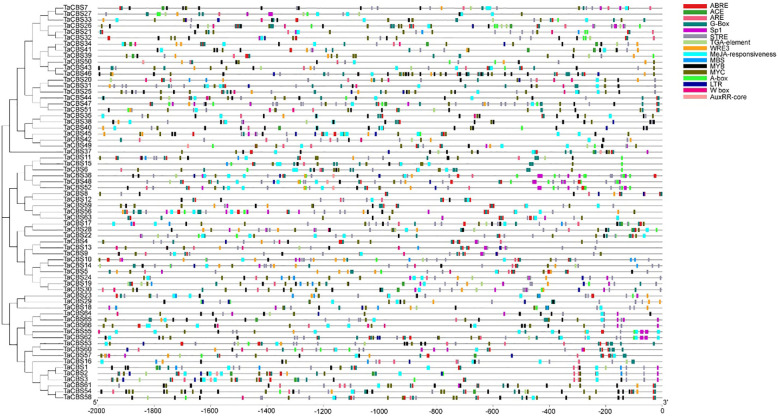
Kind, quantity and position of cis-acting elements in TaCBSs. 2000 bp nucleotide length of the gene promoter is indicated on the horizontal axis; color codes indicate different cis-acting elements. The phylogenetic tree is drawn in the same way as **Figure 2**.

In order to further understand the role of *TaCBS* genes, the 2000bp upstream of the start codon of the wheat CBS gene was selected as the promoter sequence, and the cis-acting elements in the gene promoter region were predicted online by TBtools and PlantCARE. The results showed that in addition to some traditional promoter conserved regions such as TATA-box and CAAT-box, there are many important cis-acting elements related to stress and hormone response in the upstream of wheat CBS. As shown in **Figure 6**, a total of 16 different cis elements were identified in the promoter sequences of 66 wheat CBS family genes, such as antioxidant response element (ARE), abscisic acid response element (ABRE), light responsiveness response element (ACE;G-box;SP1), cis-acting regulatory element involved in the MeJA-responsiveness (MeJA-responsiveness), stress-responsive cis-elements (STRE), wound-responsive elements (WRE3), assistent element (A-box), low-temperature responsiveness elements (LTR), the cognate cis-element for WRKY proteins elements (W box), MYB related elements (MYB, MBS), MYC elements (MYC), and auxin responsiveness elements (TGA-element, AuxRR-core) (**Figure 6**; **Supplementary Table 8**). These response elements were enriched in the promoter region of wheat *CBS* gene, suggesting that the expression of wheat *CBS* gene may respond to antioxidants, light signal stimulation, stress, and auxin signal stimulation.

### Expression pattern analysis of wheat genes encoding CDCPs in different tissues

To gain insights into the *TaCBS* expression patterns in different tissues, the RNA-seq data of wheat variety China Spring were obtained from WheatOmics 1.0. According to the expression data of the heatmap, we found genes encoding CDCPs showed different expression patterns in roots, stems, leaves, spikelets, and grains. In accordance with cluster analysis, we divided the 66 genes into two major categories: high expression region (H) and low expression region (L). These genes were further divided into 4 groups according to the branches of the evolutionary tree, namely group1 to group4.

**Figure 7:**
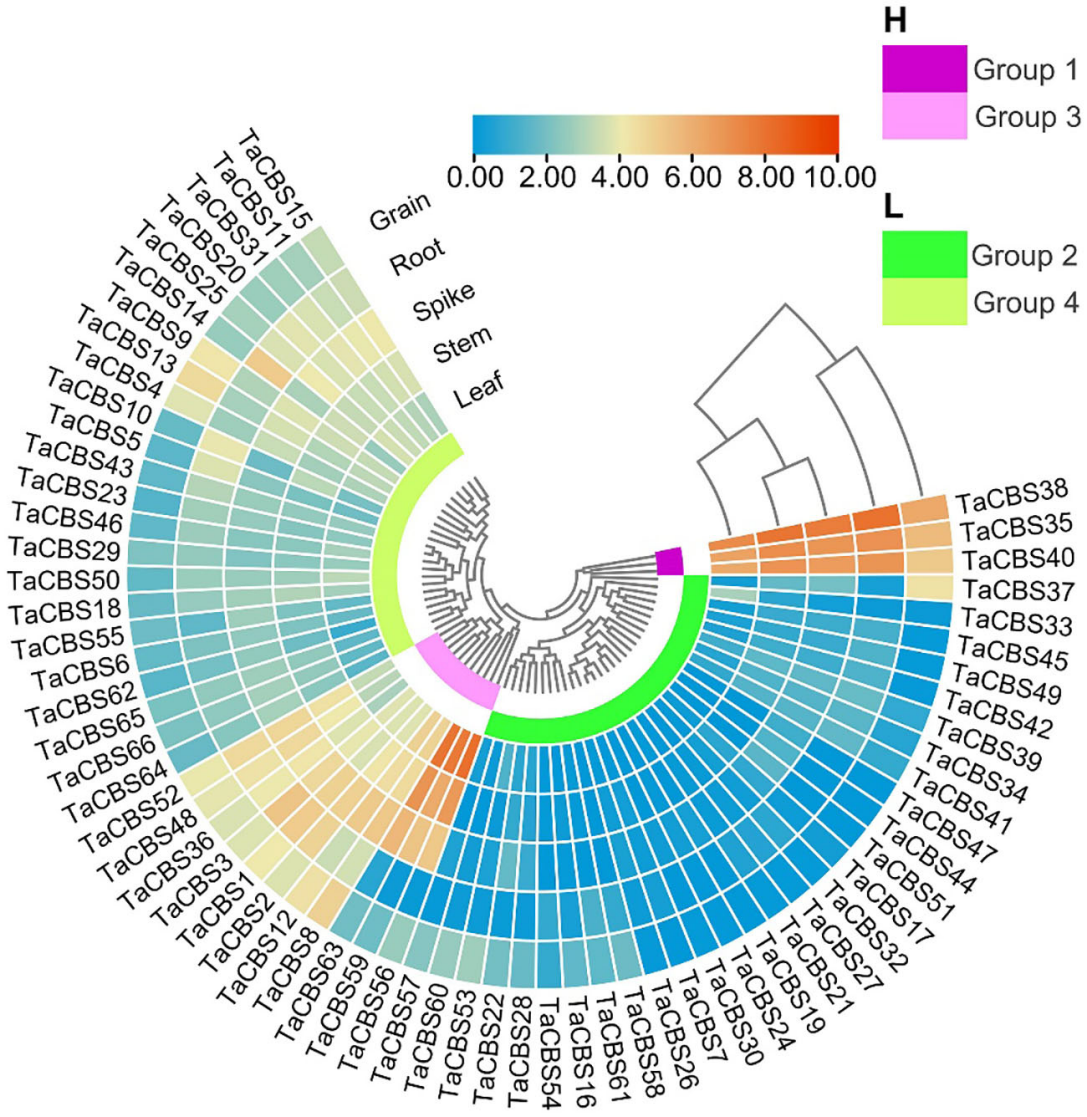
The differential expression of representative genes encoding CDCPs in different tissues by RNA-seq data reported in WheatOmics. The legend represents the log transcripts per kilobase million (TPM) values. The transcriptome expression results are shown as a heat map in blue/yellow/brownish red colors. The clusters of low and high expression are represented by different colors.

The high expression region included group1 and group3. The genes encoding CDCPs of group 1 showed high expression in all tissues, especially in roots, stems, spikelets and grains. Compared with the genes of group1, the gene expression level of group3 was lower, but the expression level of some genes in leaves and stems was higher, such as *TaCBS56*, *TaCBS59*, and *TaCBS63*. The low expression region was composed of group2 and group4. The gene expression levels of group4 were lower than those of group2, and most of these genes were expressed at low levels in various tissues. Furthermore, from the heatmap results, we can find that the expression patterns of some *TaCBS* genes in different tissues are similar, but some genes are dramatically different in various tissues. For example, the expression levels of the *TaCBS56*/*59*/*63* genes were significantly higher in the leaves than in the roots (**Figure 7**; **Supplementary Table 9**). This suggests that genes encoding CDCPs play an important role in wheat growth and development, which also implies that there may be a certain degree of biofunctional differentiation among different *TaCBS* members.

### GO and KEGG analysis of the TaCBS proteins

**Figure 8:**
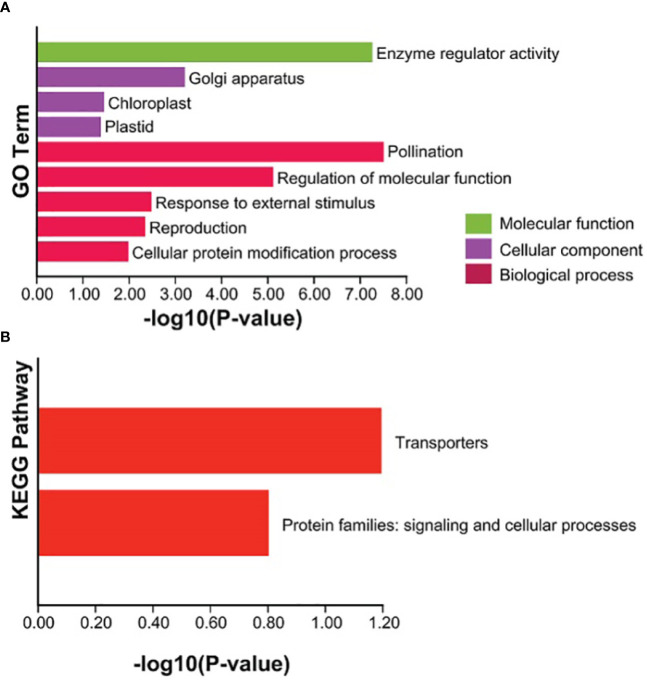
Enrichment analysis of GO and KEGG Pathway for the 66 *TaCBS* members considered in this study. **(A)** GO enrichment. Three main categories of GO enrichment are shown in green terms, purple terms, and dark red terms, respectively. **(B)** KEGG enrichment. KEGG enrichment are shown in red terms.

In order to further explore the functions of the TaCBS proteins in wheat, GO annotation was performed to analyze the cellular components, molecular functions, and biological pathway categories of these proteins. Among the cellular components, “golgi apparatus” and “chloroplast” and “plastid” are predominant. In the category of molecular functions, the TaCBS proteins have highly enriched “enzyme regulator activity”. The analysis of the biological pathway revealed that most of the TaCBS proteins were predominantly assigned to “pollination”, “regulation of molecular function”, “response to external stimulus”, “reproduction” and the “cellular protein modification process” (**Figure 8A**). Subsequently, a KEGG enrichment analysis was performed on these TaCBS proteins. “Transporters” was the most abundant KEGG enrichment in the TaCBS proteins, followed by “signaling and cellular processes” (**Figure 8B**).

### Identifying miRNA targets for *TaCBS* genes throughout the genome

**Figure 9:**
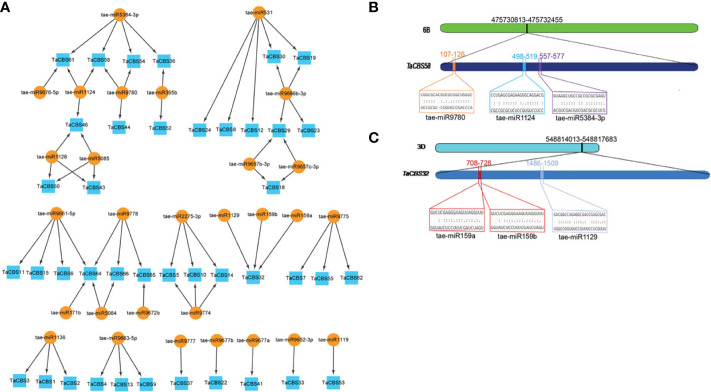
miRNAs targeting genes encoding CDCPs in wheat. **(A)** miRNA target network map for genes encoding CDCPs, with indigo boxes corresponding to *TaCBS* members and brownish yellow round shapes corresponding to predicted miRNAs. **(B)** It is evident from the graphic illustration that the *TaCBS58* gene is targeted by miRNAs (tae-miR9780, tae-miR1124, and tae-miR5384-3p). **(C)** There are three miRNAs that target the *TaCBS32* gene (tae-miR159a, tae-miR159b, and tae-miR1129) illustrated in this graphic. 6B and 3D represent chromosomes. *TaCBS58* and *TaCBS32* represent the location of miRNAs on gene sequence. Color boxes indicate the RNA sequences of the complementary sites 5’ to 3’ and the predicted miRNA sequences 3’ to 5’ in **Figures 9B, C**. The complete dataset of predicted miRNAs is presented in **Supplementary Table 10**.

In order to better understand the posttranscriptional alteration of TaCBS members by miRNAs, we identified 29 miRNAs that target 41 genes (**Figure 9A**, **Supplementary Table 10**). Based on the results, tae-miR531 targeted the largest number (5) of genes. Tae-miR5384-3p, tae-miR9666b-3p, and tae-miR9661-5p targeted four genes. While most of the miRNAs, including tae-miR1124, tae-miR9780, tae-miR1128. tae-miR5085, tae-miR9778, tae-miR2275-3p, tae-miR9774, tae-miR9775, tae-miR1136, and tae- miR9663-5p targeted three genes. There were 4 miRNAs, including tae-miR395b, tae-miR9657b-3p, tae-miR9657c-3p and tae-miR5084 targeting 2 genes. And the remaining miRNAs, including tae-miR171b, tae-miR9672b, tae-miR9777, tae-miR9677b, tae-miR9677a, tae-miR9652-3p and tae-miR1119 targeted 1 gene. Interestingly, several genes are targeted by multiple miRNAs, for example, TaCBS58, TaCBS32, TaCBS64 are targeted by three, three and four miRNAs, respectively (**Figure 9A**; **Supplementary Table 10**). To gain a better visual representation, we mapped the miRNA targeting sites of *TaCBS58* and *TaCBS32* (**Figures 9B, C**), while **Supplementary Table 10** provides all miRNA targeting sites/genes.

### The connection between wheat CBS family and anther sterility initiated by high temperature and qRT−PCR investigation

**Figure 10:**
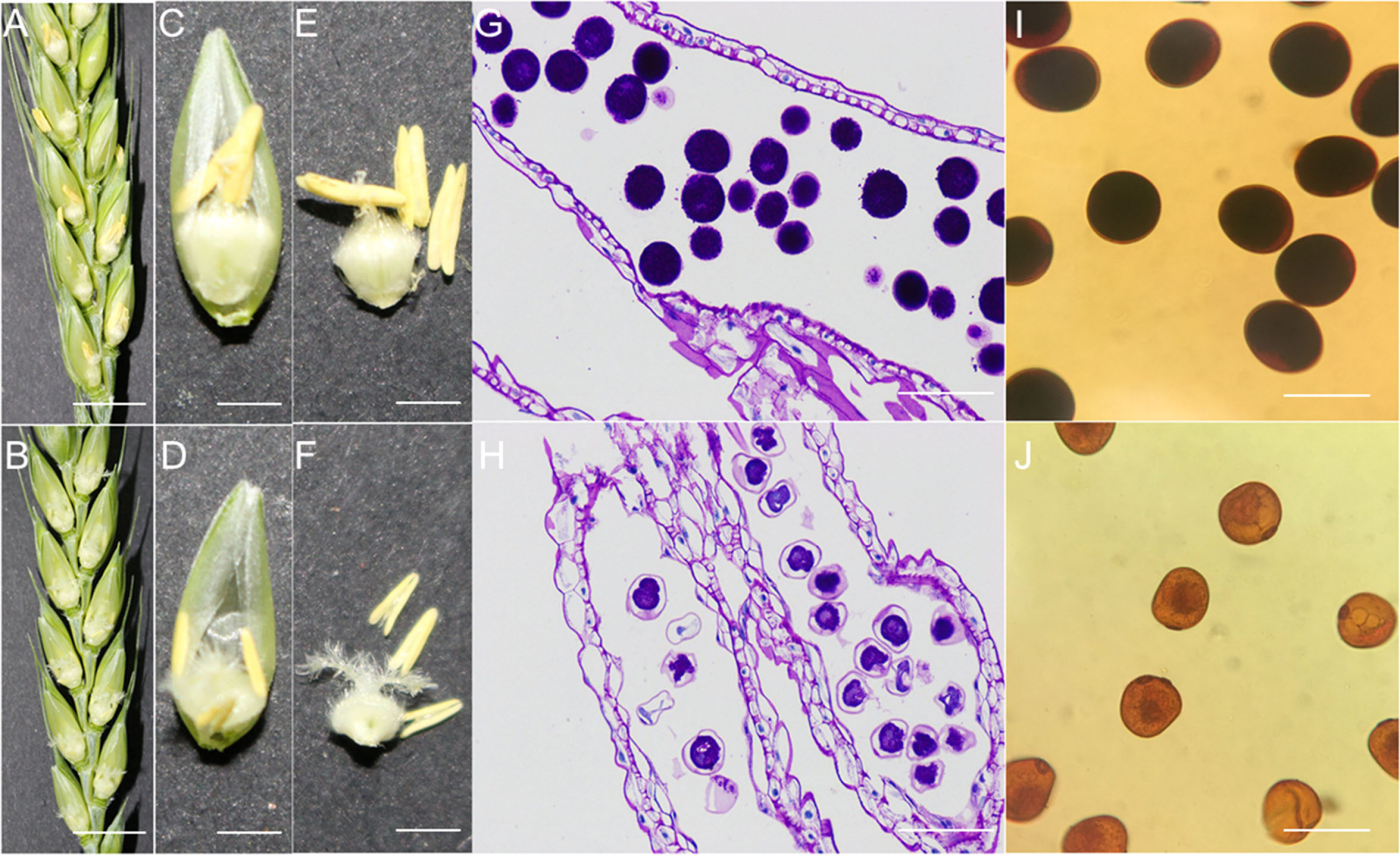
Comparison between phenotypic traits and transections of the anthers and pollen grains in Normal and HT-ms plants. **(A, C, E)** The morphology of Normal plants in anthesis on spikelets, florets and anthers, respectively. **(B, D, F)** The morphology of HT-ms plants in anthesis on spikelets, florets and anthers, respectively. **(G)** Longitudinal sections of Normal anther locule at the trinuclear stage. **(H)** Longitudinal sections of HT-ms anther locule at the trinuclear stage. **(I, J)** Pollen grains from the Normal anthers and HT-ms anthers stained with potassium iodide iodine (KI-I_2_) solution. Bars = 10mm in **(A**, **B)**, 5mm in **C-E**, **F**, and 100µm in **G-J**.

In our previous studies, it was affirmed that wheat goes through fitting heat stress during female stamen primordia development, which can prompt total male sterility in anthers. In addition, our preliminary transcriptome results revealed that one of the hub genes expressed is a CBS domain-containing protein CBSX6-like, which showed significant differences in expression between heat-stressed male sterile (HT-ms) anthers and Normal anthers, leading us to speculate that high temperature-induced male sterility in wheat may have some relationship with the *CBS* gene. Here, qRT-PCR analysis was performed to further explore the expression of *CBS* gene at multiple stages of HT-ms anthers. Phenotypic observation revealed that Normal anthers were able to extend beyond the glumes, while heat-stressed male sterile (HT-ms) anthers had no extension beyond the glumes around the feathery stigma (**Figures 10A, B**). After peeling off the individual florets, it was found that the anthers of Normal florets had pollen dispersed and the ovary had developed, while the HT-ms anthers were smaller and thinner than the Normal anthers and had no pollen dispersed, and the stigma of the ovary of sterile florets still appeared feathery and the ovary failed to develop (**Figures 10C, D**). After further stripping out the anthers and ovaries, it was found that the Normal anthers had obvious dehiscence at the top and pollen was dispersed, while the HT-ms anthers did not have dehiscence (**Figures 10E, F**). The Periodic Acid-Schiff (PAS) stained sections showed that the pollen grains of HT-ms anthers had less starch accumulation than those of Normal anthers and the epidermal layer was thinner than that of Normal anthers (**Figures 10G, H**). Staining of pollen grains with KI-I_2_ showed a complete black color in Normal anthers, but in HT-ms anthers pollen grains were of a light yellow color, meaning no starch accumulation or less starch accumulation (**Figures 10I, J**), and these results were consistent with the PAS staining sections results.

**Figure 11:**
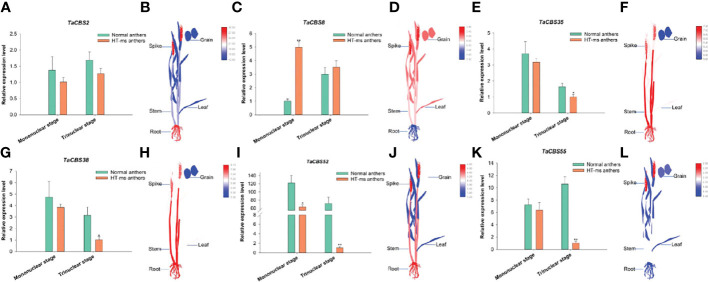
The differential expression of 6 genes encoding CDCPs in Normal and HT-ms anther tissues by qRT-PCR **(A, C, E, G, I, K)** and different tissues by RNA-seq data **(B, D, F, H, J, L)**. The x-axes and y-axes indicate the different stage in the Normal and HT-ms anthers and the relative gene expression levels, respectively. The electronic Fluorescent Pictograph (eFP) of wheat plant were visualized by Adobe Illustrator CS5 and TBtools. SPSS Statistics 23 software was used to analyze the data as means of three replicates ± standard error. Tukey’s method was used to test significantly different means between parameters based on analysis of variance (ANOVA) at 95% confidence levels. Capped lines indicate standard error. *P < 0.05; **P < 0.01.

To further explore the function of genes encoding CDCPs in wheat, we investigated the expression levels of these genes in Normal and HT-ms anthers at the mononuclear and trinuclear stages. Firstly, combining the results of synteny analysis and transcriptome expression heatmap results, we selected three genes with collinearity (*TaCBS35*, *38*, *55*) and three genes without collinearity (*TaCBS2*, *8*, *52*) for qRT-PCR analysis, respectively. Meanwhile, these genes were highly expressed in wheat spikes in the heatmap and their qRT-PCR expression is quantified in **Figure 11** (**Supplementary Tables 9**, **11** for detailed data). Compared with Normal anthers, five genes showed lower expression at the mononuclear and trinuclear stages of HT-ms anthers, especially more pronounced at the trinuclear stage; for example, genes *TaCBS52* and *TaCBS55* differed to highly significant levels. In contrast, the TaCBS8 member showed elevated expression at both the mononuclear and trinuclear stages of HT-ms anthers compared with Normal anthers (**Figure 11**). *TaCBS2* gene expression in anthers from HT-ms was 0.73-FC (fold change) lower at the mononuclear stage and 0.75-FC lower at the trinuclear stage than that of Normal anthers (**Figure 11A**). It was evident from the electronic Fluorescent Pictograph (eFP) from RNA-seq data that this gene showed a high expression status in roots (26.9 TPM) and spikelets (28.6 TPM) (**Figure 11B**). It was intriguing to note that the *TaCBS8* gene displayed a completely opposite expression pattern to *TaCBS2* at both the mononuclear and trinuclear stages. Comparing HT-ms anthers with Normal anthers, *TaCBS8* expression was 3.94-FC higher at mononuclear and 0.18-FC higher at trinuclear stages (**Figure 11C**). In addition, the eFP of this gene showed high expression in all tissues except the roots (10.9 TPM), such as in the spikelets, leaves and grains with TPM values of 35.2, 27.0 and 27.5, respectively (**Figure 11D**). At the mononuclear stage and trinuclear stage, HT-ms anthers were 0.16-FC and 0.63-FC lower than Normal anthers in terms of the *TaCBS35* gene (**Figure 11E**). Additionally, the eFP showed that this gene had relatively high expression level in the tissues of roots, stems, and spikelets (120.6 TPM, 134.0 TPM and 113.3 TPM, respectively), but low relative expression level in the tissues of grains with TPM value of 47.5 (**Figure 11F**). The expression trend of *TaCBS38* gene in anthers and in different tissues of the eFP showed a very similar expression to that of *TaCBS35* gene (**Figures 11 G, H**). *TaCBS52* gene showed extremely high expression in both mononuclear and trinuclear Normal anthers. The expression of *TaCBS52* gene was 1.93-FC and 69.77- FC higher in mononuclear and trinuclear Normal anthers than in HT-ms anthers, showing significant and extremely significant differences, respectively (**Figure 11I**). The eFP showed that this gene was relatively highly expressed in roots (23.4 TPM), stems (19.8 TPM), and spikelets (24.9), moderately expressed in grains (14.8 TPM), and was relatively low in leaves (9.0 TPM) (**Figure 11J**). For the *TaCBS55* gene, the expression of HT-ms anthers was lower than that of Normal anthers at the mononuclear stage, but did not reach a significant level, while at the trinuclear stage, the expression of HT-ms anthers was 9.27-FC lower than that of Normal anthers, showing an extremely significant difference (**Figure 11K**). The eFP based on RNA-seq data showed that this gene was relatively highly expressed in spikelets (5.4 TPM) and was relatively low in other tissues, such as in roots, leaves, and grains with TPM values of 2.0, 2.2 and 2.5, respectively (**Figure 11L**).

## Discussion

Wheat is a heterozygous polyploid with a genome composed of three very similar yet distinct sets of subgenomes A, B, and D. Heteropolyploids are natural hybrids with significant polyploidy advantages over diploid species, but the high degree of repetitive sequences and the tedious analysis of wheat genes are not comparable with other crops. In recent years, with the advancement of genome sequencing technology, wheat has had its entire genome sequenced and its genome database has become increasingly comprehensive and has been published, which can be used to identify wheat gene families, study wheat gene function, and mine wheat functional genes (Consortium, T.I.W.G.S 2014; Zhu et al., 2021). A diverse variety of biological processes are mediated by the CBS family in plants, which improves resistance to biotic and abiotic stresses and involves the process of anther development (Ali et al., 2021; Liu et al., 2021a). Thus, it is imperative to explore the functions and mechanisms of CBS proteins in wheat plants that are also subjected to biotic and abiotic stresses during growth and development. The function and characterization of CBS proteins during anther growth and male sterility induced by heat stress in wheat have not been studied until now. In this work, a total of 66 *TaCBS* genes were identified at the genome-wide level in wheat, and their gene duplication events, phylogenetic relationships were explored, and GO and KEGG analyses were systematically and comprehensively performed. In addition, to analyze and explore the variation of CBS gene expression in Normal and HT-ms anthers, we performed qRT-PCR validation.s.

Cystathionine beta synthase is one of the key enzymes involved in the methionine cycle in the metabolism of homocysteine. In many proteins, the CBS domain acts as a sensor of cellular energy metabolism and plays a regulatory role in maintaining intracellular redox (Ignoul and Eggermont, 2005). There are several studies on CBS proteins in *Arabidopsis*, rice, cotton, soybean and other crops (Wang et al., 2004; Kushwaha et al., 2009; Hao et al., 2016; Ali et al., 2021). Wang et al. found that the CBS structural domain-containing *TaCDCP1* gene in wheat is likely to be involved in the defense of wheat against stripe-inducing bacteria through the ABA signaling pathway, as well as in signal transduction pathways under low-temperature and drought environments (Wang et al., 2010). In addition, defense under low-temperature and drought conditions has also been related to studies showing that high levels of methionine can have toxic effects on plants.

From the phylogenetic tree, 66 genes could be divided into 4 subgroups, and the gene structure, structural domains, and motif characteristics of each subgroup clearly showed conserved traits (**Figure 2**). In the first subgroup, most CBS family members contain 2 to 4 CBS structural domains, but no transmembrane domains. In the second subgroup, CBS family members contain 1 or 2 CBS domains, five of the CBS proteins contain 8-9 transmembrane domains (CBSCLC), and the remaining proteins contain a relatively large number of low complexity regions. Interestingly, the third subgroup contains only 1 CBS family member with 2 CBS domains (CBSDUF1), 4 transmembrane domains, and 2 low complexity regions. The last subgroup of CBS family members contains between two and four CBS structural domains, of which six members contain only two CBS domains and with no transmembrane domains and low complexity regions; most of the remaining members contain four CBS structural domains, a Phox and Bem1p domain (CBSCBSPB1), and a transmembrane domain (**Figure S1**). It can be inferred from the differences in the number of CBS domains and the presence or absence of transmembrane domains that some functional differentiation of CBS proteins may have occurred. With the development of molecular biology techniques, the functions of CBS protein family members in the model plant *Arabidopsis* and rice have been preliminarily resolved, confirming that the CBS protein family does have diverse functions (Ramon et al., 2013; Kumar et al., 2018).

In general, as different species undergo different replicative selection events during evolution, homologous genes undergo different degrees of differentiation, and three common types are the generation of new functions, the generation of secondary functions, and the loss of replicative genes or functional redundancy (Force et al., 1999). The 66 TaCBS members in this study were unequally distributed on 21 chromosomes, with chromosomes 3A, 3B, and 3D being the most abundant (**Figure 3**). The Ka/Ks ratios were all much smaller than 1 (**Figure S2**), indicating a strong purifying selection of paralogous gene pairs in the wheat *CBS* gene family, and also suggesting that the wheat CBS gene family has stabilized during long-term evolution. In rice and cotton, the Ka/Ks ratio associated with the CBS gene family is also less than 1, which is in agreement with our results (Ali et al., 2021; Tomar et al., 2022). Furthermore, the main driving force for gene family expansion during evolution is generally thought to arise from tandem and segmental replication (Cannon et al., 2004). This study observed that duplication of chromosome segments tended to occur more frequently in wheat genome clusters with CBS gene family members in the same genomic group, such as chromosome groups 3, 5, and 6. Intergenomic segment duplications only occurred on chromosomes 4A, 4D, and 5A, 5B, and 5D (**Figure 4**). It has been found that members of gene families located in the same subgroup share common origins and conserved functions, and that the functions of paralogous or orthologous homologs can be determined from the functions of known genes (Job et al., 2018). Therefore, we constructed a comparative synthetic map of wheat with four representative species (i.e., rice, *Brachypodium distachyum*, foxtail millet, and barley) in order to further infer the phylogenetic mechanisms of the CBS gene family in wheat (**Figure 5**). There were 37 orthologous gene pairs between rice and *Brachypodium distachyum* and 34 orthologous gene pairs between millet and barley. The numbers of orthologous pairs shared between rice, wheat, and *Brachypodium distachyum* was 25. In contrast, there was only one orthologous homologous gene shared by millet, barley and wheat (**Figure 5**). These results suggest that the TaCBS members shared by these several species may have been highly conserved during evolution. For instance, the *TaCBS55* (*TraesCS6A02G283600.3*) gene is directly homologous in all five species, suggesting that this gene may have exhibited a highly conserved state during evolution. Furthermore, the expression of this gene remained convergent in roots, stems, leaves and seeds, and showed a relatively high expression in spikelets. In addition, the members of *TaCBS38* and *TaCBS35*, which are homologous pairs in all three species except barley, showed a highly (**Figure 7**).

In our previous study, it was found that male sterility induced by HT was associated with CBS protein and ROS level (Liu et al., 2021a). Recently, a study reported a novel gene *DPS1* that regulates the process of apical spikelet degeneration and reduces spike fertility, which may play a crucial role in regulating ROS homeostasis, anther cuticle formation and spike development in rice. Furthermore, they found that *DPS1* encodes a mitochondrial-localized protein containing the cystathionine β-synthase structural domain and that its expression is highest in panicles and anthers using map cloning techniques. This study also revealed that *DPS1* interacts directly with the mitochondrial thioredoxin-reducing proteins Trx1 and Trx20 and is involved in ROS scavenging. Loss-of-function mutants of *DPS1* show reduced ROS scavenging capacity, leading to ROS accumulation in *DPS*1 mutants. (Zafar et al., 2020). From the anther phenotype and paraffin sections in this study, we were able to observe three extremely distinctive features of the heat-induced sterile anthers compared with Normal anthers: First, the anthers did not extend beyond the glumes and did not exhibit dehiscence (**Figures 10A–D**). Second, they were smaller and thinner (**Figures 10E, F**). Third, the pollen grains accumulated remarkably little starch (**Figures 10G–J**). The predicted results of this study for cis-acting elements involved MeJA and auxin, which two hormones have been revealed to be strongly associated with anther indehiscence. In *Arabidopsis*, defective anther dehiscence 1 gene mutant buds were rescued by administration of exogenous JA or linolenic acid, which is consistent with reduced accumulation of JA in defective anther dehiscence 1 gene mutant buds (Ishiguro et al., 2001). In eggplant, researchers screened anther dehiscence-related genes by transcriptome sequencing and found that anther dehiscence is associated with a variety of hormones, such as IAA, GA, ABA and JA, with the most prominent decrease in JA in sterile anthers (Wang et al., 2021). In addition, in the wheat line 4110S of thermo-sensitive genic male sterility, researchers have found that high temperatures reduce the level of jasmonic acid in sterile anthers with indehiscence as their phenotypic characteristic. There may be blockages in the production of jasmonic acid, which causes anthers to fail to dehisce, eventually leading to male sterility (Yang et al., 2020). As well, a similar result was demonstrated in our previous study that high temperature significantly reduces levels of OPDA (12-oxo-phytodienoic acid) and JA-ILE (isoleucine jasmonic acid) in male sterile anthers (Liu et al., 2021b). In the present study, most of the TaCBS members identified had these JA and IAA-related cis-acting elements in the upstream region (**Figure 6**), suggesting that the expression of genes encoding CDCPs is closely related to these cis-acting elements, which may have a relationship with high temperature induced male sterility.

In the present study, a total of 29 putative tae-miRNAs have been identified, with 41 *TaCBS* members being targeted by these miRNAs. miRNAs, widespread in plant cells, are small nuclear regulatory molecules involved in many important life processes and can directly control the expression of target genes *via* post-transcriptional negative regulation (Rhoades et al., 2002; Han et al., 2021). miRNAs and target genes form a complex regulatory network. This regulatory network may finely regulate the expression of a single gene through the combination of several miRNAs, or it may regulate the expression of multiple genes through a single miRNA (Xie et al., 2010; Ding et al., 2020). There are multiple such cases in our predicted results, for example, tae-miR5384-3p and tae-miR9666b-3p have multiple target genes, while tae-miR1129, tae-miR159a, and tae-miR159b jointly regulate *TaCBS32* (**Figure 9**). One study reported that the precursor of miR167a was cloned from the photothermosensitive genic male sterile line BS366 of wheat and that overexpression of *TaemiR167a* in *Arabidopsis* led to male sterility and the expression levels of *ATARF6* and *ATARF8* were down-regulated (Wang et al., 2019). From these phenomena above and the results obtained from our predictions, it is speculated that these miRNAs, together with the wheat_*TaCBS* gene, are likely to be associated with heat stress-induced male sterility. Of course, these are subject to further experimental validation.

According to qRT-PCR results, five *TaCBS* members were under-expressed in the mononuclear and trinuclear anthers of HTms compared with Normal anthers. The *TaCBS8* member showed an elevated expression trend in both the mononuclear and trinuclear stages of sterile anthers compared with the same period in Normal anthers, especially showing a highly significant difference in the mononuclear stage (**Figure 11**). In terms of gene structure, this gene has a remarkably long intron sequence with two CBS domains and a low complexity region (**Figure 2**; **Figure S1**), and the different expression trend of this gene from other genes may be related to its own gene structure. Another example is that the members TaCBS35 and TaCBS38 had almost identical expression trends (**Figure 11**), and their motifs and gene structures were found to be extremely similar by analysis, and the evolutionary trees were clustered to the same branch (**Figure 2**). And to go further, the TaCBS8 member is not involved in synteny, whereas the TaCBS35 and TaCBS38 members are a pair of paralogous homologs and are orthologous homologous to other species (**Figure 4**), which indirectly suggests that the functions of these genes may have diverged somewhat. Additionally, the heatmap showed that all these genes showed high expression in spikelet tissues based on the RNA-seq data (**Figure 11**). In *Arabidopsis*, by regulating Trx, CBSX1 participates in anther dehiscence; CBSX2 regulates JA and JA signaling in the floral organ, while JA, in response to MYB transcription factors, leads to thickening of the endothelial cell wall and thus regulates anther dehiscence, which researchers speculate is important to ensure flower fertility and eventual seed production (Yoo et al., 2011; Jung et al., 2013). In rice, researchers have found that *DPS1* encodes a protein containing the structural domain of cysteine β-synthase and hypothesized that *DPS1* plays a role in male reproduction and seed setting (Zafar et al., 2020). Similarly, the down-regulation of the majority of genes encoding CDCPs in the expression of HT-ms anthers in the present study suggests an association of genes encoding CDCPs with sterility caused by high-temperature induction in wheat. 

## Conclusions

In this study, we identified 66 genes encoding CDCPs in wheat. Based on protein motifs, gene structure, chromosomal location, Ka/Ks analysis, cis-acting elements, putative miRNAs analysis, synteny analysis and expression pattern analysis, TaCBS members are conservative and diversified. Here, small sizes and indehiscence are the distinguishing characteristics of HT-ms anthers, and the cis-elements predicted in the first 2000bp of our identified *TaCBS* gene sequence contained promoter elements related to anther development or dehiscence such as MeJA, IAA, and MYB. Twenty-nine miRNAs targeting 41 TaCBS members were identified, and the analysis of the regulatory relationships between these miRNAs and *TaCBS* gene interactions further increased our understanding of *TaCBS* genes. Moreover, the qRT-PCR analysis revealed that HT-ms anthers expressed a significantly lower level of *TaCBS2*, *TaCBS35*, *TaCBS38*, *TaCBS52*, and *TaCBS55* at trinuclear stage than Normal anthers. These outcomes indicate that the wheat gene family encoding CDCPs may have some relationship with high temperature-induced male sterility. In addition, the abnormal expression of these *TaCBS* members may be one of the reasons why HT-ms anthers develop small and with no dehiscence, which may be one of the factors that eventually lead to another abortion.

## Data availability statement

The datasets presented in this study can be found in online repositories. The names of the repository/repositories and accession number(s) can be found in the article/**Supplementary Material**.

## Author contributions

HL and GT designed research and conducted experiments, HL performed data analysis and wrote the manuscript. QW, LX, KX, FZ, XR, and LL helped to collect anthers and improve the method and edited the manuscript. KX, FZ, and GT revised the manuscript. All authors read and approved the manuscript.
