# Corrigendum: Genome-wide identification of genes encoding cystathionine beta synthase domain-containing proteins in wheat and its relationship with anther male sterility under heat stress

**DOI:** 10.3389/fpls.2023.1198918

**Published:** 2023-06-01

**Authors:** Hongzhan Liu, Qi Wang, Liuyong Xie, Kedong Xu, Fuli Zhang, Xianle Ruan, Lili Li, Guangxuan Tan

**Affiliations:** ^1^ College of Life Science and Agronomy, Zhoukou Normal University, Zhoukou, Henan, China; ^2^ School of Network Engineering, Zhoukou Normal University, Zhoukou, Henan, China; ^3^ Key Laboratory of Plant Genetics and Molecular Breeding, Zhoukou Normal University, Zhoukou, Henan, China; ^4^ Institute of Plant Protection and Edible Mushrooms, Zhoukou Academy of Agricultural Sciences, Zhoukou, Henan, China

**Keywords:** *Triticum aestivum* L., gene repetition events, cystathionine beta synthase, synteny analysis, gene expression

In the published article, there was an error in the article title. Instead of “*Genome-wide identification of cystathionine beta synthase genes in wheat and its relationship with anther male sterility under heat stress*”, it should be “*Genome-wide identification of genes encoding cystathionine beta synthase domain-containing proteins in wheat and its relationship with anther male sterility under heat stress*”.

In the published article, there was an error in the legend for

**Table 1**, As requested by the reviewer, we have added an explanation of these genes in the footnotes of **Table 1**. The corrected legend and footnote appear below.

“**Table 1**. Information about the *TaCBS* members in wheat.

**Table 1:** Information about the TaCBS members in wheat.

Gene Name	Gene Locus	CDS Length (bp)	AA^a^	MW^b^ (kDa)	pI^c^	TMD^d^	SLP^e^
*TaCBS1*	TraesCS1A02G170000.1	1122	373	38.88	5.35	0	mitochondrion
*TaCBS2*	TraesCS1B02G187200.1	1122	373	38.95	5.47	0	mitochondrion
*TaCBS3*	TraesCS1D02G167600.1	1122	373	38.93	5.47	0	mitochondrion
*TaCBS4*	**TraesCS2A02G177000.1**	1362	453	48.06	4.82	0	cytoplasm
	TraesCS2A02G177000.2	1035	344	36.28	5.36	0	cytoplasm
*TaCBS6*	TraesCS2A02G281700.1	1278	425	46.95	5.45	0	nucleus
*TaCBS6*	TraesCS2A02G289200.1	1047	348	38.64	5.92	0	nucleus
	**TraesCS2A02G289200.2**	1371	456	50.92	5.56	0	nucleus
	TraesCS2A02G289200.3	1365	454	50.68	5.56	0	nucleus
	TraesCS2A02G289200.4	1314	437	48.82	5.58	0	nucleus
*TaCBS7*	TraesCS2A02G360600.1	1515	504	54.71	6.61	0	chloroplast
*TaCBS8*	TraesCS2B02G002100.1	666	221	23.38	7.87	0	chloroplast thylakoid membrane
*TaCBS9*	TraesCS2B02G203900.1	1260	419	44.39	4.97	0	cytoplasm
	**TraesCS2B02G203900.2**	1362	453	47.96	4.82	0	cytoplasm
	TraesCS2B02G203900.3	1149	382	40.74	5.00	0	nucleus
*TaCBS10*	TraesCS2B02G299000.1	1278	425	46.91	5.62	0	nucleus
*TaCBS11*	TraesCS2B02G305800.1	1050	349	38.69	6.00	0	nucleus
	TraesCS2B02G305800.2	1239	412	45.59	5.50	0	extracellular space
	TraesCS2B02G305800.3	1185	394	43.76	6.15	0	nucleus
	TraesCS2B02G305800.4	1371	456	50.89	5.74	0	nucleus
	TraesCS2B02G305800.5	1365	454	50.65	5.74	0	nucleus
	**TraesCS2B02G305800.6**	1395	464	51.83	5.74	0	nucleus
	TraesCS2B02G305800.7	1068	355	39.38	5.83	0	nucleus
	TraesCS2B02G305800.8	1164	387	43.34	6.76	0	nucleus
	TraesCS2B02G305800.9	1032	343	38.22	5.64	0	extracellular space
*TaCBS12*	TraesCS2D02G015500.1	666	221	23.52	8.73	0	chloroplast thylakoid membrane
	**TraesCS2D02G015500.2**	672	223	23.94	8.71	0	chloroplast thylakoid lumen
*TaCBS13*	**TraesCS2D02G185000.1**	1362	453	48.00	4.82	0	cytoplasm
	TraesCS2D02G185000.2	1035	344	36.33	5.23	0	cytoplasm
*TaCBS14*	TraesCS2D02G280600.1	1278	425	46.95	5.45	0	nucleus
*TaCBS15*	TraesCS2D02G287200.1	1047	348	38.70	5.65	0	nucleus
	TraesCS2D02G287200.2	1365	454	50.76	5.45	0	nucleus
	TraesCS2D02G287200.3	1068	355	39.48	5.42	0	nucleus
	**TraesCS2D02G287200.4**	1371	456	51.00	5.45	0	nucleus
	TraesCS2D02G287200.5	1164	387	43.45	6.51	0	nucleus
*TaCBS16*	TraesCS2D02G599900.1	1149	382	39.76	4.92	0	nucleus
*TaCBS17*	TraesCS3A02G226700.1	1206	401	44.09	5.67	0	nucleus
*TaCBS18*	TraesCS3A02G427100.1	1197	398	42.18	6.01	0	nucleus
*TaCBS19*	TraesCS3A02G429700.1	1290	429	46.99	5.14	0	nucleus
*TaCBS20*	TraesCS3A02G433400.1	1125	374	40.66	6.23	1	endomembrane system
	**TraesCS3A02G433400.2**	1653	550	58.85	7.25	1	nucleus
*TaCBS21*	TraesCS3A02G445200.1	1632	543	58.45	6.10	1	chloroplast
*TaCBS22*	TraesCS3B02G257800.1	1209	402	44.21	5.70	0	nucleus
*TaCBS23*	TraesCS3B02G463800.1	1197	398	42.21	5.93	0	nucleus
*TaCBS24*	TraesCS3B02G467600.1	1323	440	47.71	5.22	0	nucleus
*TaCBS25*	TraesCS3B02G469200.1	1125	374	40.62	6.22	1	endomembrane system
	**TraesCS3B02G469200.2**	1650	549	58.75	7.24	1	nucleus
*TaCBS26*	TraesCS3B02G479900.1	1392	463	49.86	5.02	1	chloroplast
*TaCBS27*	TraesCS3B02G573900.1	1128	375	40.53	5.42	0	nucleus
*TaCBS28*	TraesCS3D02G224700.1	1218	405	44.19	5.45	0	nucleus
*TaCBS29*	TraesCS3D02G422700.1	1197	398	42.23	5.93	0	nucleus
*TaCBS30*	TraesCS3D02G425000.1	1293	430	47.07	5.20	0	nucleus
*TaCBS31*	TraesCS3D02G426800.1	1656	551	59.03	7.25	1	nucleus
*TaCBS32*	TraesCS3D02G438100.1	1632	543	58.54	6.23	1	chloroplast
*TaCBS33*	**TraesCS3D02G513700.1**	1578	525	56.14	6.77	1	chloroplast
*TaCBS34*	**TraesCS4A02G206600.1**	1635	544	58.23	6.40	1	chloroplast
*TaCBS35*	TraesCS4A02G247300.1	618	205	22.40	9.14	0	mitochondrion
*TaCBS36*	TraesCS4A02G320000.1	1494	497	54.40	6.25	0	nucleus
	TraesCS4A02G320000.2	1467	488	53.55	6.34	0	nucleus
*TaCBS37*	TraesCS4B02G022400.1	1464	487	54.21	6.42	4	endomembrane system
*TaCBS38*	TraesCS4B02G067600.1	642	213	23.39	9.10	0	mitochondrion
*TaCBS39*	TraesCS4B02G110100.1	1632	543	58.02	6.25	1	chloroplast
	**TraesCS4B02G110100.2**	1764	587	63.71	5.81	0	nucleus
*TaCBS40*	TraesCS4D02G066600.1	618	205	22.40	9.14	0	mitochondrion
*TaCBS41*	TraesCS4D02G107800.1	1635	544	58.17	6.58	1	chloroplast
*TaCBS42*	TraesCS5A02G053000.1	618	205	23.05	7.92	0	mitochondrion
*TaCBS43*	TraesCS5A02G118800.1	1635	544	58.79	6.78	1	chloroplast
*TaCBS44*	TraesCS5A02G209500.1	1623	540	58.71	6.59	1	mitochondrion
*TaCBS45*	TraesCS5B02G063400.1	618	205	23.10	8.65	0	mitochondrion
*TaCBS46*	TraesCS5B02G117400.1	1464	487	52.77	6.30	0	chloroplast
	**TraesCS5B02G117400.2**	1641	546	59.02	7.70	1	chloroplast
*TaCBS47*	TraesCS5B02G207600.1	1626	541	58.75	6.40	1	mitochondrion
*TaCBS48*	**TraesCS5B02G559100.1**	1494	497	54.31	6.05	0	nucleus
	TraesCS5B02G559100.2	1467	488	53.49	6.14	0	nucleus
*TaCBS49*	TraesCS5D02G064300.1	618	205	23.05	7.92	0	mitochondrion
*TaCBS50*	**TraesCS5D02G130200.1**	1629	542	58.49	6.82	1	chloroplast
	TraesCS5D02G130200.2	1452	483	52.28	6.13	0	chloroplast
*TaCBS51*	TraesCS5D02G215700.1	1626	541	58.81	8.40	1	mitochondrion
*TaCBS52*	TraesCS5D02G565200.1	1125	374	41.04	6.60	0	extracellular space
	TraesCS5D02G565200.2	1467	488	53.59	6.31	0	nucleus
	**TraesCS5D02G565200.3**	1494	497	54.42	6.22	0	nucleus
*TaCBS53*	TraesCS6A02G132700.1	1317	438	46.55	5.17	0	nucleus
*TaCBS54*	TraesCS6A02G235600.1	1176	391	40.70	5.41	0	mitochondrion
*TaCBS55*	TraesCS6A02G283600.1	1791	596	63.61	6.06	7	endomembrane system
	**TraesCS6A02G283600.3**	2346	781	83.05	6.77	9	organelle membrane
*TaCBS56*	TraesCS6A02G392100.1	651	216	23.59	8.64	0	chloroplast
	**TraesCS6A02G392100.2**	654	217	23.72	8.64	0	chloroplast
	TraesCS6A02G392100.3	636	211	23.11	8.30	0	chloroplast
*TaCBS57*	TraesCS6B02G160900.1	1311	436	46.51	5.11	0	nucleus
*TaCBS58*	TraesCS6B02G264200.1	1167	388	40.53	5.35	0	nucleus
*TaCBS59*	TraesCS6B02G432300.1	654	217	23. 41	7.62	0	nucleus
	**TraesCS6B02G432300.2**	657	218	23.54	7.62	0	nucleus
	TraesCS6B02G432300.3	606	201	21.57	6.89	0	chloroplast
*TaCBS60*	TraesCS6D02G122400.1	1323	440	46.85	5.37	0	nucleus
*TaCBS61*	TraesCS6D02G218300.1	1158	385	40.07	5.43	0	nucleus
*TaCBS62*	TraesCS6D02G264100.1	2358	785	83.62	6.59	9	organelle membrane
*TaCBS63*	TraesCS6D02G378000.1	651	216	23.49	8.31	0	chloroplast
	TraesCS6D02G378000.2	636	211	23.01	7.64	0	chloroplast
	**TraesCS6D02G378000.3**	747	248	26.92	6.22	0	nucleus
	TraesCS6D02G378000.4	606	201	21.77	7.66	0	chloroplast
*TaCBS64*	TraesCS7A02G240700.2	2232	743	79.25	6.19	8	endomembrane system
*TaCBS65*	TraesCS7B02G136300.1	2232	743	79.11	6.19	8	endomembrane system
*TaCBS66*	TraesCS7D02G239700.2	2232	743	79.23	6.10	8	endomembrane system
	**TraesCS7D02G239700.3**	2241	746	79.55	6.49	8	endomembrane system

^a^Length of the amino acid sequence. ^b^Molecular weight of the amino acid sequence. ^c^Isoelectric point of the TaCBS proteins. ^d^Number of transmembrane domains, as predicted by the TMHMM server. ^e^Protein subcellular localization prediction by the BUSCA web server. The bold values indicate the selected transcript representing this gene. All *TaCBS* genes in the table indicate genes encoding cystathionine beta synthase domain-containing proteins.

^a^Length of the amino acid sequence. ^b^Molecular weight of the amino acid sequence. ^c^Isoelectric point of the TaCBS proteins. ^d^Number of transmembrane domains, as predicted by the TMHMM server. ^e^Protein subcellular localization prediction by the BUSCA web server. The bold values indicate the selected transcript representing this gene. All *TaCBS* genes in the table indicate genes encoding cystathionine beta synthase domain-containing proteins.”

In the published article, there was an error in the legend for **Figure 3**, As requested by the reviewer, we have modified the legend of **Figure 3**. The corrected legend appears below.

**Figure 2:**
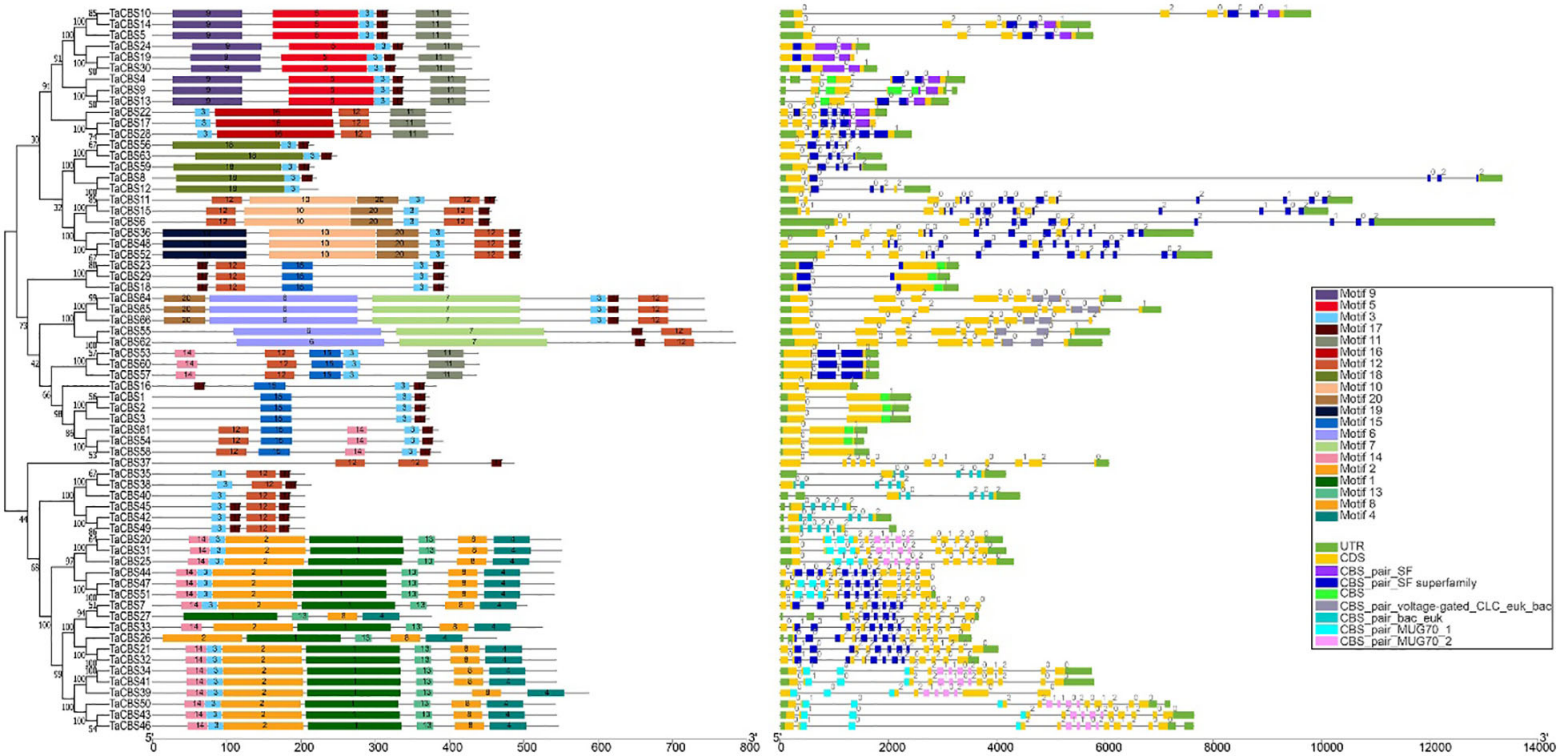
Phylogenetic relationship, conserved motifs, gene structure, and conserved domain of TaCBS proteins. Different colors are used to represent different motifs in the figure and in the upper right corner. Black lines represent non-conserved sequences in MEME results and introns in the exon-intron structure, respectively. The phylogenetic tree is constructed similarly to Figure 1.

**Figure 3:**
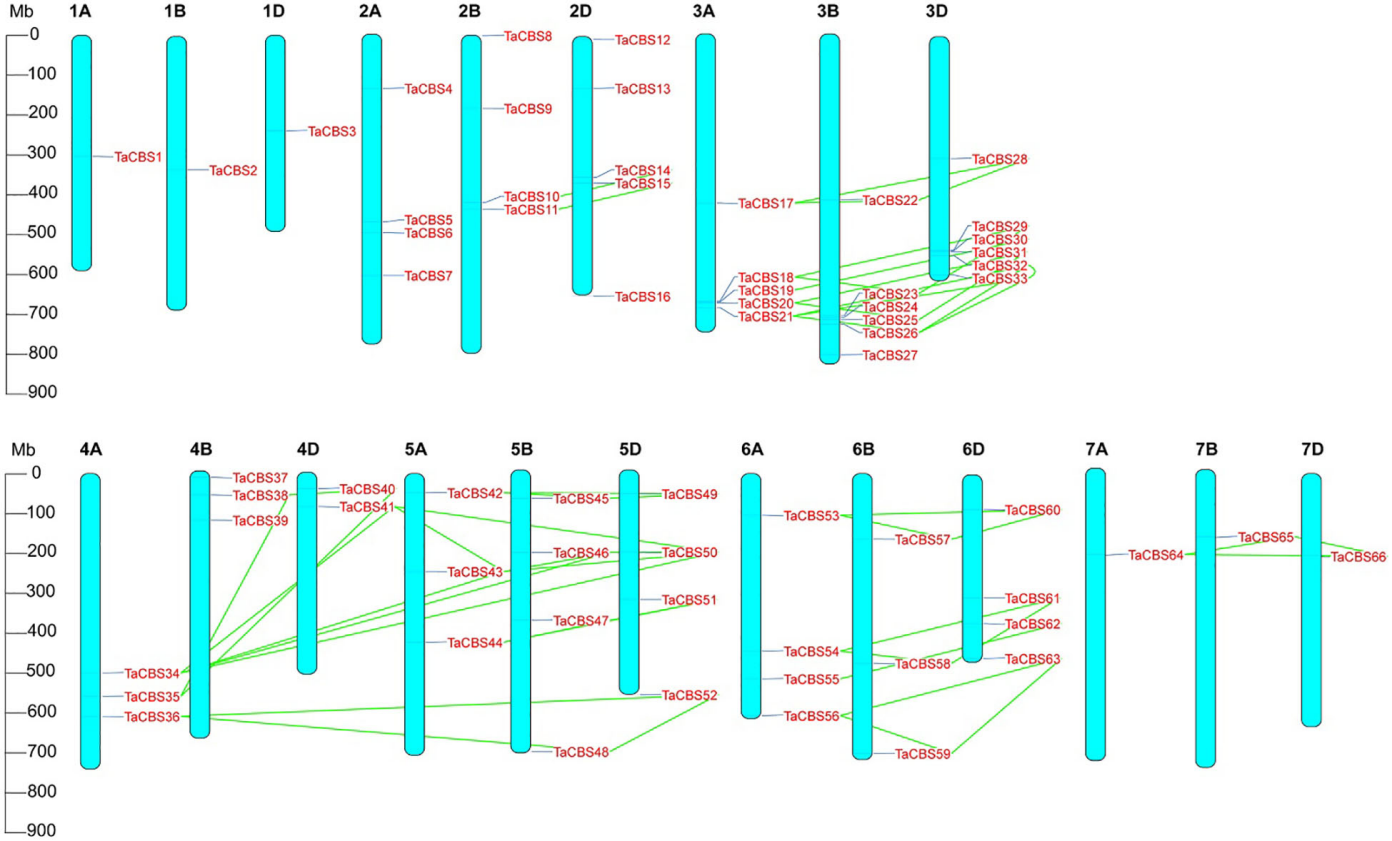
Chromosomal localization of the genes encoding CDCPs in the wheat genome. The positions on the linkage map have been determined. 66 TaCBS members are mapped to 21 chromosomes (1A-7A, 1B-7B, and 1D-7D). Green colored lines represent the connections between duplication events. Megabase pairs (Mb) are used to measure the scale.

“**Figure 3** Chromosomal localization of the genes encoding CDCPs in the wheat genome. The positions on the linkage map have been determined. 66 *TaCBS* members are mapped to 21 chromosomes (1A-7A, 1B-7B, and 1D-7D). Green colored lines represent the connections between duplication events. Megabase pairs (Mb) are used to measure the scale.”

In the published article, there was an error in the legend for **Figure 4**. As requested by the reviewer, we have modified the legend of **Figure 4**. The corrected legend appears below.

**Figure 4:**
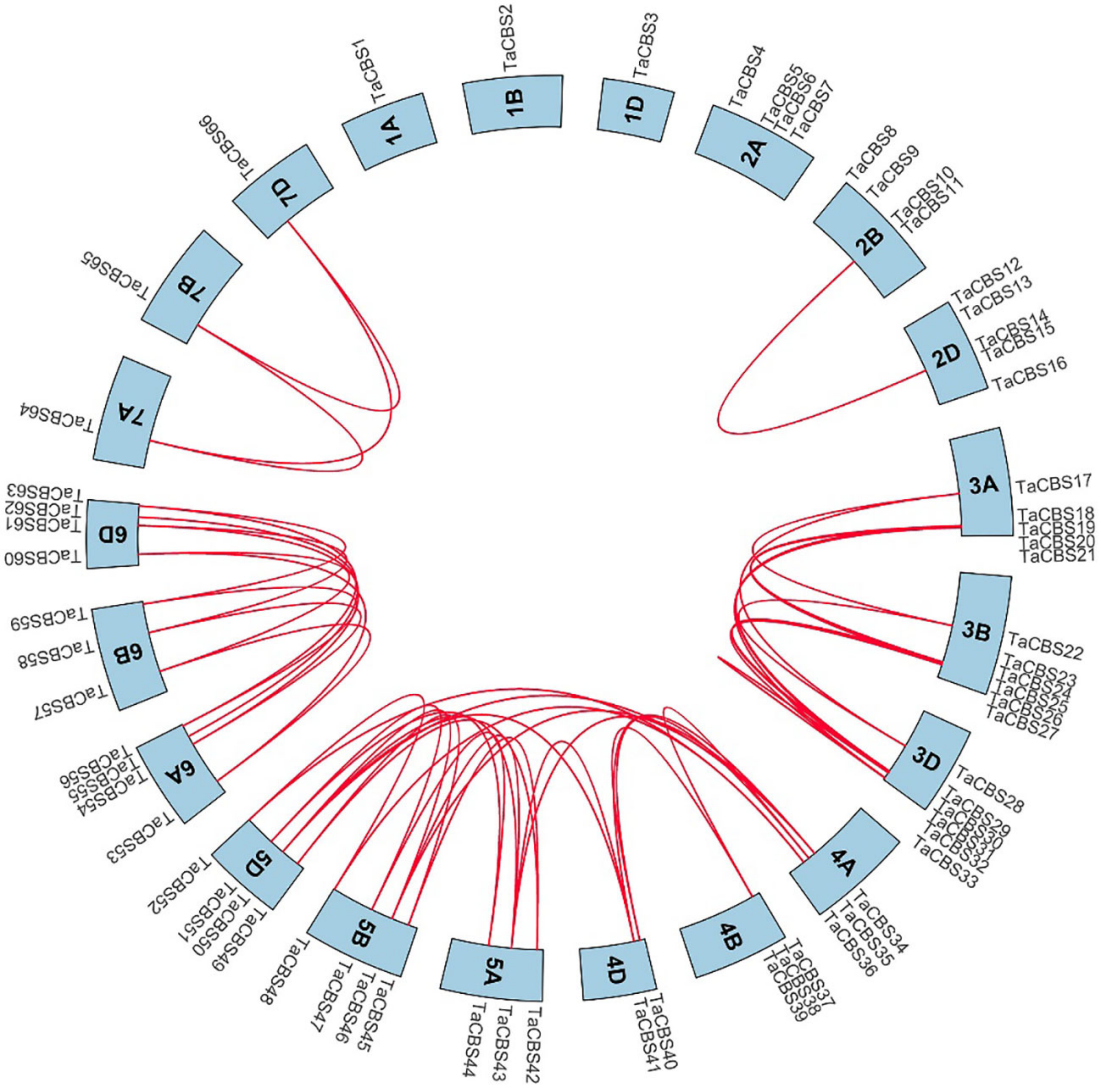
Genome-wide syntenic and localization analysis of the genes encoding CDCPs in wheat. The genes encoding CDCPs in wheat were mapped to different chromosomes with a cyan color. The red line indicates the synteny of gene pairs within the *TaCBS*. On the chromosome’s outermost side are the *TaCBS* members names.

“**Figure 4** Genome-wide syntenic and localization analysis of the genes encoding CDCPs in wheat. The genes encoding CDCPs in wheat were mapped to different chromosomes with a cyan color. The red line indicates the synteny of gene pairs within the *TaCBS*. On the chromosome’s outermost side are the *TaCBS* members names.”

In the published article, there was an error in the legend for **Figure 7**. As requested by the reviewer, we have modified the first sentence of the legend of **Figure 7**. The corrected legend appears below.

**Figure 6:**
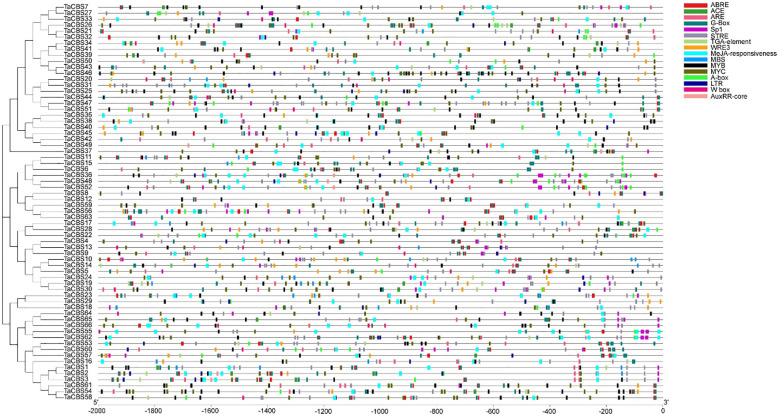
Kind, quantity and position of cis-acting elements in TaCBSs. 2000 bp nucleotide length of the gene promoter is indicated on the horizontal axis; color codes indicate different cis-acting elements. The phylogenetic tree is drawn in the same way as **Figure 2**.

**Figure 7:**
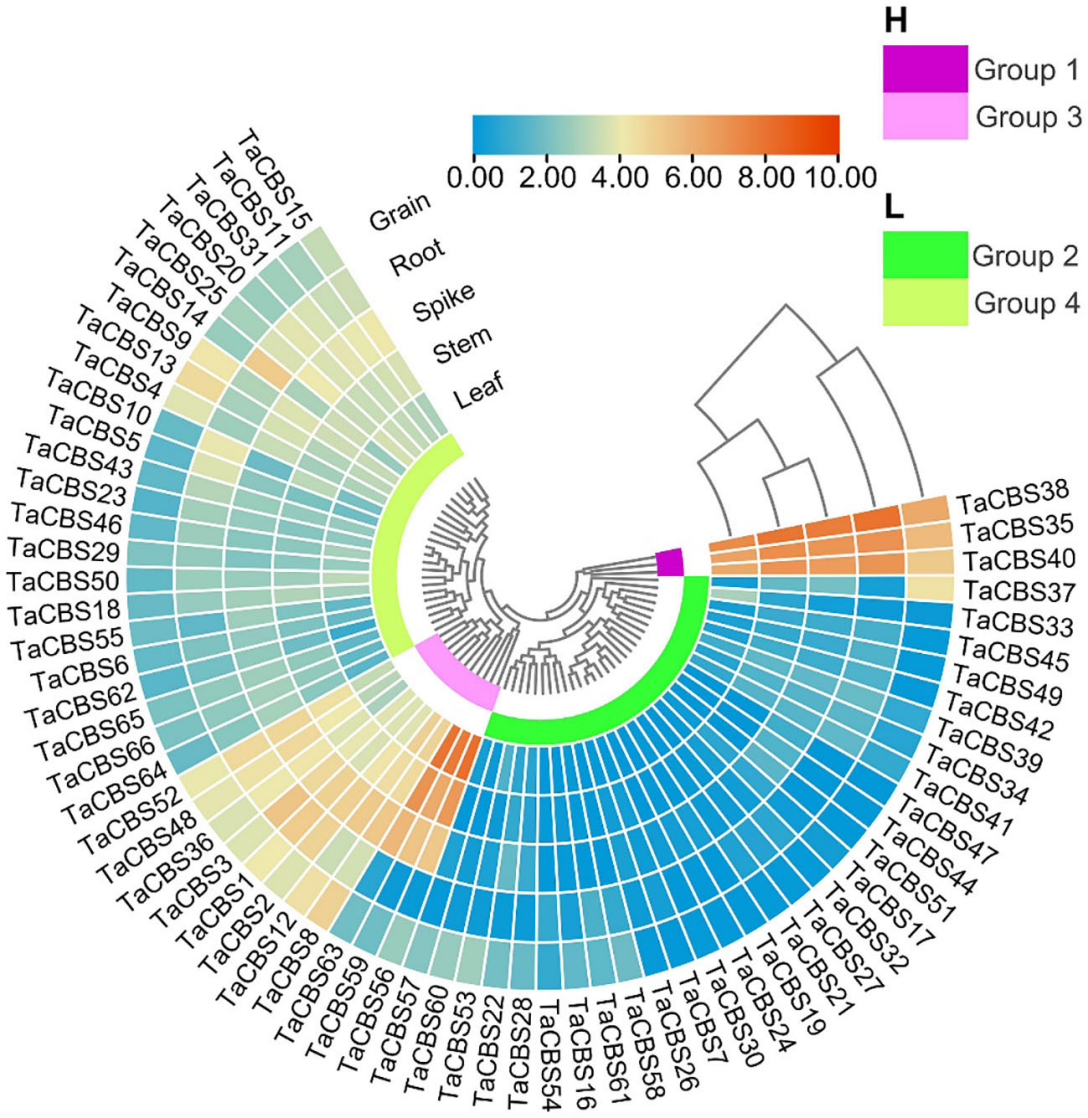
The differential expression of representative genes encoding CDCPs in different tissues by RNA-seq data reported in WheatOmics. The legend represents the log transcripts per kilobase million (TPM) values. The transcriptome expression results are shown as a heat map in blue/yellow/brownish red colors. The clusters of low and high expression are represented by different colors.

“**Figure 7** The differential expression of representative genes encoding CDCPs in different tissues by RNA-seq data reported in WheatOmics.”

In the published article, there was an error in the legend for **Figure 8**. As requested by the reviewer, we have modified the first sentence of the legend of **Figure 8**.The corrected legend appears below.

**Figure 8:**
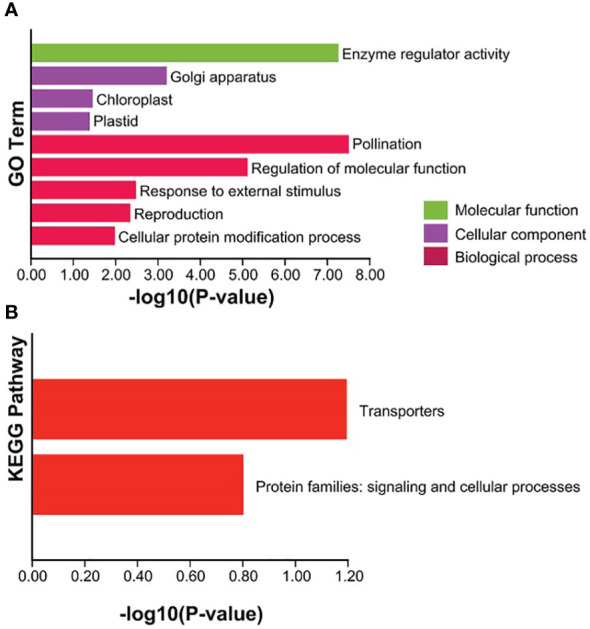
Enrichment analysis of GO and KEGG Pathway for the 66 TaCBS members considered in this study. **(A)** GO enrichment. Three main categories of GO enrichment are shown in green terms, purple terms, and dark red terms, respectively. **(B)** KEGG enrichment. KEGG enrichment are shown in red terms.

“**Figure 8** Enrichment analysis of GO and KEGG Pathway for the 66 TaCBS members considered in this study.”

In the published article, there was an error in the legend for **Figure 9**. As requested by the reviewer, we have modified the first and second sentences of the legend of **Figure 9**. The corrected legend appears below.

**Figure 9:**
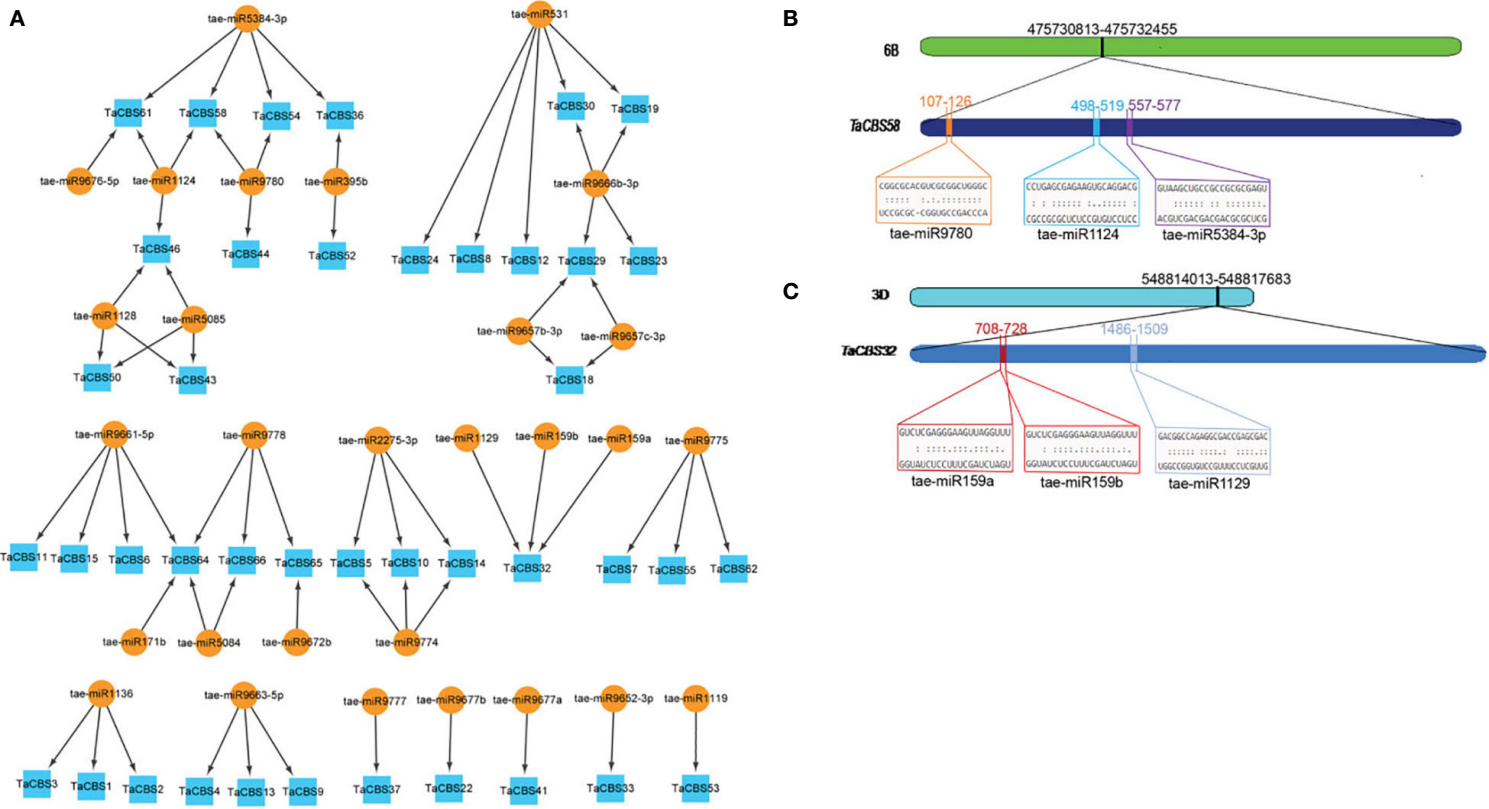
miRNAs targeting genes encoding CDCPs in wheat. **(A)** miRNA target network map for genes encoding CDCPs, with indigo boxes corresponding to *TaCBS* members and brownish yellow round shapes corresponding to predicted miRNAs. **(B)** It is evident from the graphic illustration that the *TaCBS58* gene is targeted by miRNAs (tae-miR9780, tae-miR1124, and tae-miR5384-3p). **(C)** There are three miRNAs that target the *TaCBS32* gene (tae-miR159a, tae-miR159b, and tae-miR1129) illustrated in this graphic. 6B and 3D represent chromosomes. *TaCBS58* and *TaCBS32* represent the location of miRNAs on gene sequence. Color boxes indicate the RNA sequences of the complementary sites 5’ to 3’ and the predicted miRNA sequences 3’ to 5’ in **Figures 9B, C**. The complete dataset of predicted miRNAs is presented in **Supplementary Table 10**.

“**Figure 9** miRNAs targeting genes encoding CDCPs in wheat. (A) miRNA target network map for genes encoding CDCPs, with indigo boxes corresponding to *TaCBS* members and brownish yellow round shapes corresponding to predicted miRNAs.”

In the published article, there was an error in the legend for **Figure 11**. As requested by the reviewer, we have modified the legend of **Figure 11**. The corrected legend appears below.

**Figure 11:**
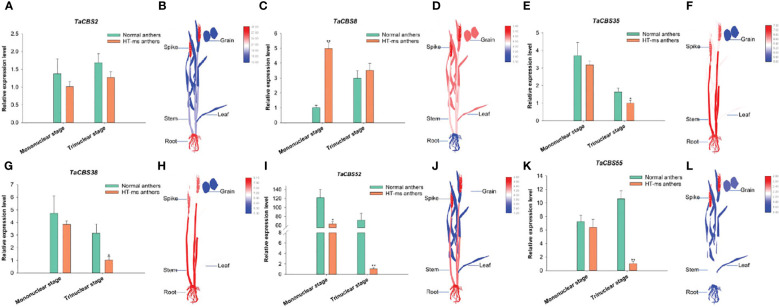
The differential expression of 6 genes encoding CDCPs in Normal and HT-ms anther tissues by qRT-PCR **(A, C, E, G, I, K)** and different tissues by RNA-seq data **(B, D, F, H, J, L)**. The x-axes and y-axes indicate the different stage in the Normal and HT-ms anthers and the relative gene expression levels, respectively. The electronic Fluorescent Pictograph (eFP) of wheat plant were visualized by Adobe Illustrator CS5 and TBtools. SPSS Statistics 23 software was used to analyze the data as means of three replicates±standard error. Tukey's method was used to test significantly different means between parameters based on analysis of variance (ANOVA) at 95% confidence levels. Capped lines indicate standard error. *P < 0.05; **P < 0.01.

“**Figure 11** The differential expression of 6 genes encoding CDCPs in Normal and HT-ms anther tissues by qRT-PCR (A, C, E, G, I, and K) and different tissues by RNA-seq data (B, D, F, H, J, and L). The x-axes and y-axes indicate the different stage in the Normal and HT-ms anthers and the relative gene expression levels, respectively. The electronic Fluorescent Pictograph (eFP) of wheat plant were visualized by Adobe Illustrator CS5 and TBtools. SPSS Statistics 23 software was used to analyze the data as means of three replicates ± standard error. Tukey’s method was used to test significantly different means between parameters based on analysis of variance (ANOVA) at 95% confidence levels. Capped lines indicate standard error. *P < 0.05; **P < 0.01”.

In the published article, there was an error in the **Supplementary Material**. As requested by the reviewer, we have modified **Figure S1** and the legend of **Figure S1** within Presentation 1. The correct figure and legend appears below.

**“Figure S1** Results of classification and conserved domains predicted using the SMART web site.

The CBS domain, the transmembrane region, the Phox and Bem1 (PB1) domain and the complexity region are represented by the pentagonal element, the blue column, the triangular element and the pink rectangular element, respectively. Other domains include DUF21, voltage chloride channel (Voltage CLC), and Carbohydrate binding domain (CBD). The classification according to one pair or two pairs of CBS domains (CBSX and CBSCBS). Other classifications are named by the other structural domains they contain for labeling, such as CBSCLC, CBSCBSCBD, CBSDUF1 and CBSCBSPB1. The 66 identified members corresponding to this protein are shown in parentheses.”

A correction has been made to the **Abstract**. As requested by the reviewer, we have modified the text. This sentence previously stated:

“Together with anther phenotypes, paraffin sections, starch potassium iodide staining, and qRT-PCR data, we hypothesized that the *TaCBS* gene has a very important connection with the heat-stressed sterility process in wheat, and these data provide a basis for further insight into their relationship.”

The corrected sentence appears below:

“Together with anther phenotypes, paraffin sections, starch potassium iodide staining, and qRT-PCR data, we hypothesized that the genes encoding CDCPs has a very important connection with the heat-stressed sterility process in wheat, and these data provide a basis for further insight into their relationship.”

A correction has been made to **Introduction**, paragraph 4. As requested by the reviewer, we have modified the text.

These sentences previously stated:

“In addition, research on the CBS gene family has mainly concentrated on soybean, rice, cotton and Arabidopsis (Kushwaha et al., 2009; Ali et al., 2021; Hao et al., 2021), but there is no report on wheat. Therefore, considering the above studies, we speculate that CBS gene may play an important role in regulating fertility, especially in indehiscence of sterile anther in wheat, and these issues are worthy of further exploration. In this study, the genome-wide members of the wheat CBS gene family were identified by bioinformatics methods, and the physiological and biochemical properties, conserved motifs, cis-elements, gene collinearity and gene expression patterns of all family members were comprehensively analyzed. qRT-PCR was performed for the expression patterns of 6 TaCBS genes in wheat anther indehiscence under high-temperature stress conditions. These results provide a theoretical basis and technical reference for further analysis of the functional roles of the CBS gene family in wheat male sterility.”

The corrected sentence appears below:

“In addition, research on the gene family encoding CDCPs has mainly concentrated on soybean, rice, cotton and *Arabidopsis* (Kushwaha et al., 2009; Ali et al., 2021; Hao et al., 2021), but there is no report on wheat. Therefore, considering the above studies, we speculate that genes encoding CDCPs may play an important role in regulating fertility, especially in indehiscence of sterile anther in wheat, and these issues are worthy of further exploration. In this study, the genome-wide members of the wheat gene family encoding CDCPs were identified by bioinformatics methods, and the physiological and biochemical properties, conserved motifs, cis-elements, gene collinearity and gene expression patterns of all family members were comprehensively analyzed. qRT-PCR was performed for the expression patterns of 6 *TaCBS* genes in wheat anther indehiscence under high-temperature stress conditions. These results provide a theoretical basis and technical reference for further analysis of the functional roles of the gene family encoding CDCPs in wheat male sterility.”

A correction has been made to **Materials and Methods**, **Phylogenetic tree analysis of wheat CBS gene family** heading. The heading has been corrected to **Phylogenetic tree analysis of wheat gene family encoding CDCPs**.

A correction has been made in **Materials and methods, Phylogenetic tree analysis of wheat gene family encoding CDCPs**. As requested by the reviewer, we have modified the text.

This sentence previously stated:

“MEGA-X software was used to construct a phylogenetic tree of the CBS gene family from the above plant species and wheat.”

“The corrected sentence appears below:

“MEGA-X software was used to construct a phylogenetic tree of the gene family encoding CDCPs from the above plant species and wheat.”

A correction has been made to the **Materials and methods**, **Analysis of protein domain, exon and intron structure, and conserved motif of CBS gene family** heading. This heading has been corrected to **Analysis of protein domain, exon and intron structure, and conserved motif of CDCPs encoded by gene family**.

A correction has been made to the **Materials and methods**, **Cis-element prediction of CBS gene family** heading. The heading has been corrected to **Cis−element prediction of gene family encoding CDCPs**.

A correction has been made to the **Materials and methods**, **Transcriptomic data analysis of wheat CBS gene family** heading. The heading has been corrected to **Transcriptomic data analysis of wheat gene family encoding CDCPs**.

In the published article, there was in error in **Materials and methods, Transcriptomic data analysis of wheat gene family encoding CDCPs**.

The sentences previously stated:

“TBtools software was used to perform log normalization on the downloaded TPM (Transcripts Per Kilobase Million) values, and to draw the expression heatmap of wheat CBS genes.”

The corrected sentence appears below:

“TBtools software was used to perform log normalization on the downloaded TPM (Transcripts Per Kilobase Million) values, and to draw the expression heatmap of wheat genes encoding CDCPs.”

A correction has been made to the **Materials and methods**, **Prediction of putative miRNAs targeting TaCBS genes** heading. The heading has been corrected to **Prediction of putative miRNAs targeting genes encoding CDCPs**.

A correction has been made in **Materials and methods, Prediction of putative miRNAs targeting genes encoding CDCPs**.

This sentence previously stated:

“The miRNA target network map for *TaCBS* genes was generated by Cytoscape_v3.9.1 (Shannon et al., 2003).”

The corrected sentence appears below:

“The miRNA target network map for genes encoding CDCPs was generated by Cytoscape_v3.9.1 (Shannon et al., 2003).”

A correction has been made to **Results**, **Identification characterization and phylogenetic analysis of CBS genes in wheat** heading. The heading has been corrected to **Identification, characterization and phylogenetic analysis of genes encoding CDCPs in wheat.**

A correction has been made to **Results**, **Sequence features of gene structure, motifs and conserved domains of the TaCBS gene family**, paragraph 1.

These sentences previously stated:

“The phylogenetic tree of 66 *TaCBS* gene family proteins showed that these proteins can bedivided into two major branches;”

“Intron phase 0 is present in all 66 *TaCBS* genes, with 8 being the most (such as *TaCBS11*-*TaCBS52* on the phylogenetic tree (**Figure 2**).”

“According to the classification results, 66 TaCBS proteins were classified into 6 classes, namely CBSX (25 proteins), CBSCBS (11proteins), CBSCBSCBD (6 proteins), CBSCLC (5 proteins), CBSDUF1 (1protein) and CBSCBSPB1 (18 proteins). More details are available in **Figure S1** and **Supplementary Table 3**.”

The corrected sentences appear below:

“The phylogenetic tree of 66 CDCPs showed that these proteins can be divided into two major branches;”

“Intron phase 0 is present in all 66 *TaCBS* members, with 8 being the most (such as *TaCBS11*-*TaCBS52* on the phylogenetic tree (**Figure 2**)”.

“According to the classification results, 66 TaCBS proteins were classified into 6 classes, namely CBSX1-25 (25 proteins), CBSCBS1-11 (11proteins), CBSCBSCBD1-6 (6 proteins), CBSCLC1-5 (5 proteins), CBSDUF1 (1protein) and CBSCBSPB1-1-18 (18 proteins). More details are available in **Figure S1** and **Supplementary Table 3**.”

A correction has been made to **Results**, **Chromosome distribution and gene duplication of wheat TaCBS gene family**, paragraph 1.

These sentences previously stated:

“The distribution and density of *TaCBS* genes are uneven on 21 wheat chromosomes. Chromosomal localization of *TaCBS* genes is displayed in **Figure 3**, which shows that most TaCBS genes were tandemly distributed. There are 1 (in 1A, 1B, 1D, 7A, 7B, 7D), 2 (in 4D), 3 (in 4A, 4B, 5A, 6B), 4 (in 2A, 2B, 5B, 5D, 6A, 6D), 5 (in 2D, 3A) and 6 (in 3B, 3D) TaCBS genes in different chromosomes, respectively.”

“These homologous genes have homologous sites on three or two partial homologous chromosomes, indicating that the wheat TaCBS gene has a large number of homologous sites, showing a high homology retention rate. The conserved positions of these fragment replication regions located on different chromosomes suggests that fragment replication events play an important role in the expansion of the number of TaCBS genes in wheat.”

The corrected sentences appear below:

“The distribution and density of genes encoding CDCPs are uneven on 21 wheat chromosomes. Chromosomal localization of genes encoding CDCPs is displayed in **Figure 3**, which shows that most *TaCBS* members were tandemly distributed. There are 1 (in 1A, 1B, 1D, 7A, 7B, 7D), 2 (in 4D), 3 (in 4A, 4B, 5A, 6B), 4 (in 2A, 2B, 5B, 5D, 6A, 6D), 5 (in 2D, 3A) and 6 (in 3B, 3D) *TaCBS* members in different chromosomes, respectively.”

“These homologous genes have homologous sites on three or two partial homologous chromosomes, indicating that the wheat genes encoding CDCPs has a large number of homologous sites, showing a high homology retention rate. The conserved positions of these fragment replication regions located on different chromosomes suggests that fragment replication events play an important role in the expansion of the number of *TaCBS* members in wheat.”

A correction has been made to the **Results**, **Localization and synteny of the TaCBS genes in the wheat genome** heading. The heading has been corrected to **Localization and synteny of the genes encoding CDCPs in the wheat genome.**

A correction has been made to **Results, Localization and synteny of the genes encoding CDCPs in the wheat genome,** paragraph 1.

These sentences previously stated:

“We analyzed the collinearity of these TaCBS genes in the wheat genome by using the Bio-linux system with the two-way blast comparison analysis and the MCScanX tool, and a total of 52 pairs of collinearity genes were identified.”

The corrected sentence appears below:

“We analyzed the collinearity of these genes encoding CDCPs in the wheat genome by using the Bio-linux system with the two-way blast comparison analysis and the MCScanX tool, and a total of 52 pairs of collinearity genes were identified.”

A correction has been made to **Results**, **Strong purifying selection for the TaCBS gene pairs in wheat**.

This sentence previously stated:

“A further analysis of these replicated gene pairs revealed the Ka/Ks values of all wheat TaCBS genes were less than 1, with a maximum value of 0.451.”

The corrected sentence appears below:

“A further analysis of these replicated gene pairs revealed the Ka/Ks values of all wheat *TaCBS* members were less than 1, with a maximum value of 0.451.”

A correction has been made to **Results**, **Synteny analysis of TaCBS genes between wheat and four representative pplant species** heading. The heading has been corrected to **Synteny analysis of genes encoding CDCPs between wheat and four representative plant species.**

A correction has been made to the **Results**, **Expression pattern analysis of wheat CBS gene in different tissues** heading. The heading has been corrected to **Expression pattern analysis of wheat genes encoding CDCPs in different tissues**.

A correction has been made to **Results, Expression pattern analysis of wheat genes encoding CDCPs in different tissues**, paragraph 1.

This sentence previously stated:

“According to the expression data of the heatmap, we found *TaCBS* genes showed different expression patterns in roots, stems, leaves, spikelets, and grains.”

The corrected sentence appears below:

“According to the expression data of the heatmap, we found genes encoding CDCPs showed different expression patterns in roots, stems, leaves, spikelets, and grains.”

A correction has been made to **Results, Expression pattern analysis of wheat genes encoding CDCPs in different tissues**, paragraph 2.

These sentences previously stated:

“The *TaCBS* genes of group 1 showed high expression in all tissues, especially in roots, stems, spikelets and grains.”

“This suggests that *TaCBS* genes play an important role in wheat growth and development, which also implies that there may be a certain degree of biofunctional differentiation among different TaCBS members.”

The corrected sentences appear below:

“The genes encoding CDCPs of group 1 showed high expression in all tissues, especially in roots, stems, spikelets and grains.”

“This suggests that genes encoding CDCPs play an important role in wheat growth and development, which also implies that there may be a certain degree of biofunctional differentiation among different TaCBS members.”

A correction has been made to **Results**, **Identifying miRNA targets for TaCBS genes throughout the genome,** paragraph 1, These sentences previously stated:

“In order to better understand the posttranscriptional alteration of TaCBS genes by miRNAs, we identified 29 miRNAs that target 41 genes (**Figure 9A**; **Supplementary Table 10**).”

The corrected sentence appears below:

“In order to better understand the posttranscriptional alteration of *TaCBS* members by miRNAs, we identified 29 miRNAs that target 41 genes (**Figure 9A**, **Supplementary Table 10**).”

A correction has been made to **Results**, **The connection between wheat CBS family and another sterility initiated by high temperature and qRT-PCR investigation**, paragraph 2.

These sentences previously stated:

“To further explore the function of *TaCBS* gene in wheat, we investigated the expression levels of *TaCBS* gene in Normal and HT-ms anthers at the mononuclear and trinuclear stages.”

“In contrast, the *TaCBS8* gene showed elevated expression at both the mononuclear and trinuclear stages of HT-ms anthers compared with Normal anthers (**Figure 11**).”

The corrected sentence appears below:

“To further explore the function of genes encoding CDCPs in wheat, we investigated the expression levels of these genes in Normal and HT-ms anthers at the mononuclear and trinuclear stages.”

“In contrast, the *TaCBS8* member showed elevated expression at both the mononuclear and trinuclear stages of HT-ms anthers compared with Normal anthers (**Figure 11**).”

A correction has been made to **Discussion**, paragraph 4.

These sentences previously stated:

“The 66 CBS genes in this study were unequally distributed on 21 chromosomes, with chromosomes 3A, 3B, and 3D being the most abundant (**Figure 3**).”

“These results suggest that the TaCBS genes shared by these several species may have been highly conserved during evolution.”

“In addition, the genes of *TaCBS38* and *TaCBS35*, which are homologous pairs in all three species except barley, showed a highly expressed state in roots, stems, leaves, spikelets, and grains (**Figure 7**).”

The corrected sentences appear below:

“The 66 *TaCBS* members in this study were unequally distributed on 21 chromosomes, with chromosomes 3A, 3B, and 3D being the most abundant (**Figure 3**).”

“These results suggest that the *TaCBS* members shared by these several species may have been highly conserved during evolution.”

“In addition, the members of *TaCBS38* and *TaCBS35*, which are homologous pairs in all three species except barley, showed a highly expressed state in roots, stems, leaves, spikelets, and grains (**Figure 7**).”

A correction has been made to **Discussion**, paragraph 5.

This sentence previously stated:

“In the present study, most of the CBS genes identified had these JA and IAA-related cis-acting elements in the upstream region (**Figure 6**), suggesting that the expression of CBS genes is closely related to these cis-acting elements, which may have a relationship with high temperature induced male sterility.”

The corrected sentence appears below:

“In the present study, most of the *TaCBS* members identified had these JA and IAA-related cis-acting elements in the upstream region (**Figure 6**), suggesting that the expression of genes encoding CDCPs is closely related to these cis-acting elements, which may have a relationship with high temperature induced male sterility.”

A correction has been made to **Discussion**, paragraph 6.

This sentence previously stated:

“In the present study, a total of 29 putative tae-miRNAs have been identified, with 41 *TaCBS* genes being targeted by these miRNAs.”

The corrected sentence appears below:

“In the present study, a total of 29 putative tae-miRNAs have been identified, with 41 *TaCBS* members being targeted by these miRNAs.”

A correction has been made to **Discussion**, paragraph 7.

These sentences previously stated:

“According to qRT-PCR results, five CBS genes were underexpressed in the mononuclear and trinuclear anthers of HT-ms compared with Normal anthers. The TaCBS8 gene showed an elevated expression trend in both the mononuclear and trinuclear stages of sterile anthers compared with the same period in Normal anthers, especially showing a highly significant difference in the mononuclear stage (**Figure 11**).”

“Another example is that the genes *TaCBS35* and *TaCBS38* had almost identical expression trends (**Figure 11**), and their motifs and gene structures were found to be extremely similar by analysis, and the evolutionary trees were clustered to the same branch (**Figure 2**). And to go further, the TaCBS8 gene is not involved in synteny, whereas the TaCBS35 and TaCBS38 genes are a pair of paralogous homologs and are orthologous homologous to other species (**Figure 4**), which indirectly suggests that the functions of these genes may have diverged somewhat.”

“Similarly, the down-regulation of the majority of *TaCBS* genes in the expression of HT-ms anthers in the present study suggests an association of CBS genes with sterility caused by high-temperature induction in wheat.”

The corrected sentences appear below:

“According to qRT-PCR results, five *TaCBS* members were under-expressed in the mononuclear and trinuclear anthers of HT-ms compared with Normal anthers. The *TaCBS8* member showed an elevated expression trend in both the mononuclear and trinuclear stages of sterile anthers compared with the same period in Normal anthers, especially showing a highly significant difference in the mononuclear stage (**Figure 11**).”

“Another example is that the members *TaCBS35* and *TaCBS38* had almost identical expression trends (**Figure 11**), and their motifs and gene structures were found to be extremely similar by analysis, and the evolutionary trees were clustered to the same branch (**Figure 2**). And to go further, the *TaCBS8* member is not involved in synteny, whereas the *TaCBS35* and *TaCBS38* members are a pair of paralogous homologs and are orthologous homologous to other species (**Figure 4**), which indirectly suggests that the functions of these genes may have diverged somewhat.”

“Similarly, the down-regulation of the majority of genes encoding CDCPs in the expression of HT-ms anthers in the present study suggests an association of genes encoding CDCPs with sterility caused by high-temperature induction in wheat.”

A correction has been made to **Conclusions**.

These sentences previously stated:

“In this study, we identified 66 CBS genes in wheat.”

“Based on protein motifs, gene structure, chromosomal location, Ka/Ks analysis, cis-acting elements, putative miRNAs analysis, synteny analysis and expression pattern analysis, *TaCBS* genes are conservative and diversified.”

“Twenty-nine miRNAs targeting 41 *TaCBS* genes were identified, and the analysis of the regulatory relationships between these miRNAs and *TaCBS* gene interactions further increased our understanding of *TaCBS* genes.”

“These outcomes indicate that the wheat CBS gene family may have some relationship with high temperature-induced male sterility.”

“In addition, the abnormal expression of these CBS genes may be one of the reasons why HT-ms anthers develop small and with no dehiscence, which may be one of the factors that eventually lead to anther abortion.”

The corrected sentences appear below:

“In this study, we identified 66 genes encoding CDCPs in wheat.”

“Based on protein motifs, gene structure, chromosomal location, Ka/Ks analysis, cis-acting elements, putative miRNAs analysis, synteny analysis and expression pattern analysis, *TaCBS* members are conservative and diversified.”

“Twenty-nine miRNAs targeting 41 *TaCBS* members were identified, and the analysis of the regulatory relationships between these miRNAs and *TaCBS* gene interactions further increased our understanding of *TaCBS* genes.”

“These outcomes indicate that the wheat gene family encoding CDCPs may have some relationship with high temperature-induced male sterility.”

“In addition, the abnormal expression of these *TaCBS* members may be one of the reasons why HT-ms anthers develop small and with no dehiscence, which may be one of the factors that eventually lead to another abortion.”

The authors apologize for these errors and state that these do not change the scientific conclusions of the article in any way. The original article has been updated.

