# Supplementary material for: Genome-wide identification of genes encoding cystathionine beta synthase domain-containing proteins in wheat and its relationship with anther male sterility under heat stress
Source: Front Plant Sci. 2022 Dec 14;13:1061472. doi: 10.3389/fpls.2022.1061472 (PMC9795209; doi:10.3389/fpls.2022.1061472)
Supplement: Supplementary file 2 [file Presentation_1.pptx]

## Slide 1
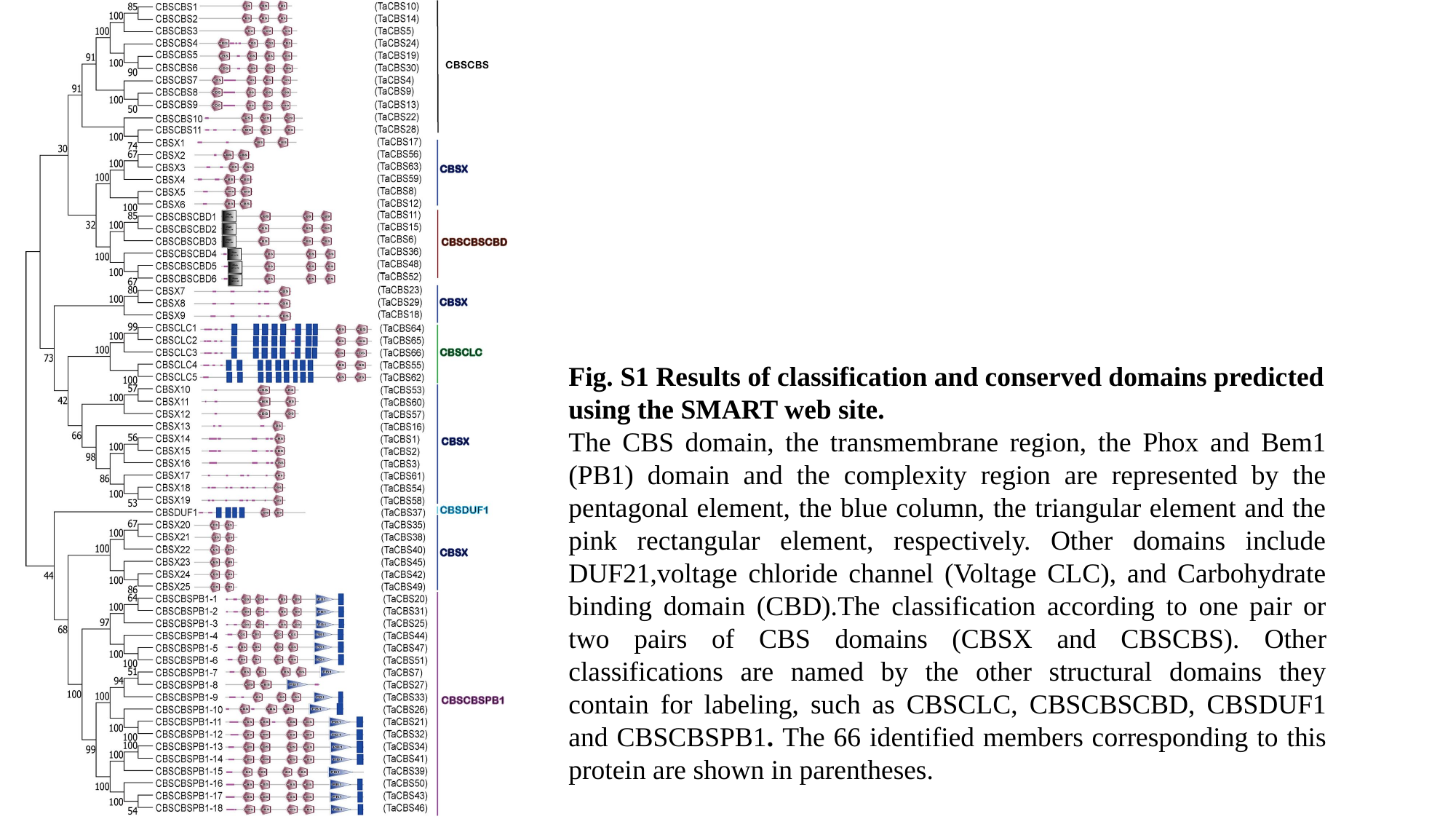

Fig. S1 Results of classification and conserved domains predicted using the SMART web site.
The CBS domain, the transmembrane region, the Phox and Bem1 (PB1) domain and the complexity region are represented by the pentagonal element, the blue column, the triangular element and the pink rectangular element, respectively. Other domains include DUF21,voltage chloride channel (Voltage CLC), and Carbohydrate binding domain (CBD).The classification according to one pair or two pairs of CBS domains (CBSX and CBSCBS). Other classifications are named by the other structural domains they contain for labeling, such as CBSCLC, CBSCBSCBD, CBSDUF1 and CBSCBSPB1. The 66 identified members corresponding to this protein are shown in parentheses.

## Slide 2
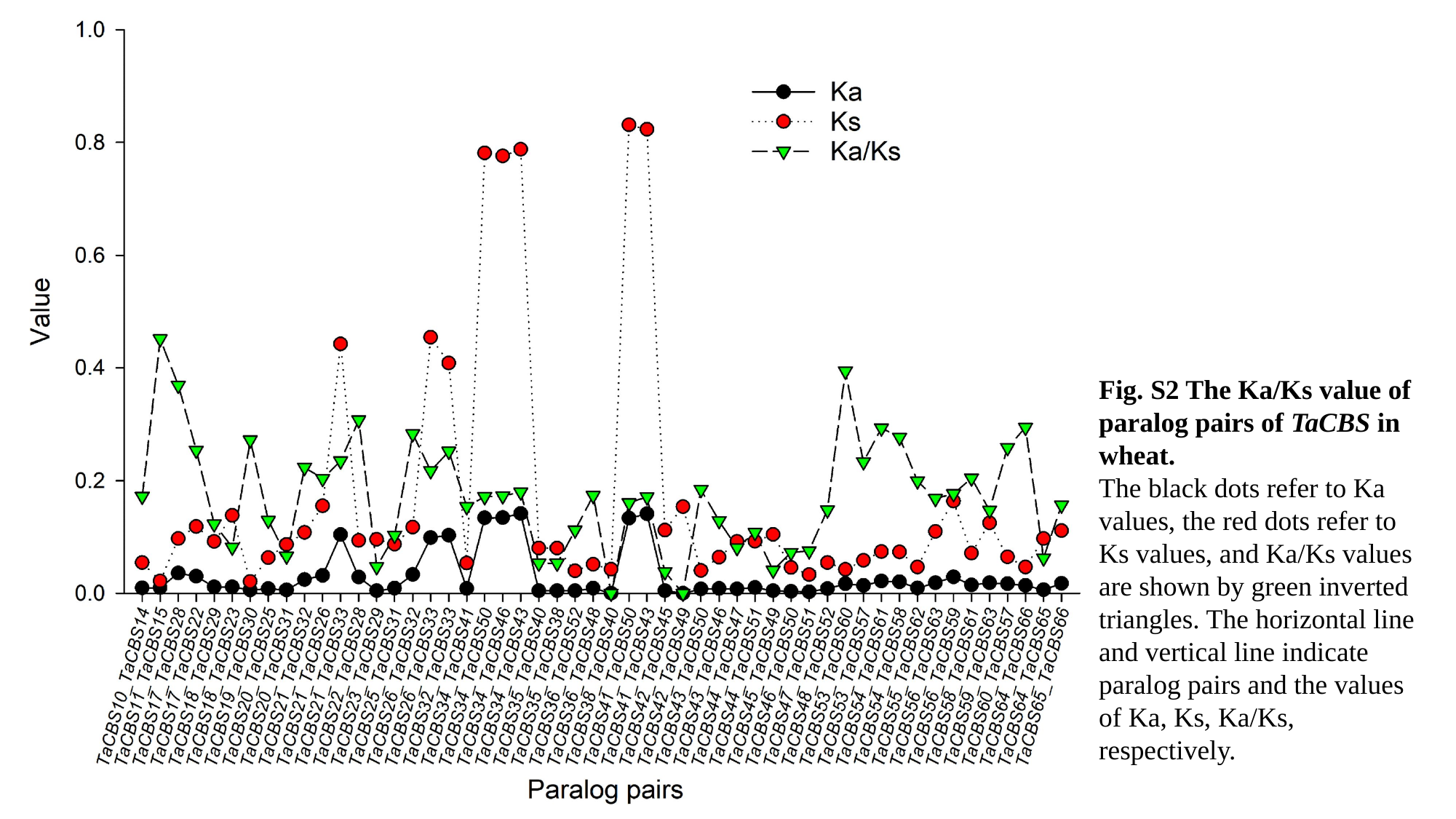

Fig. S2 The Ka/Ks value of paralog pairs of TaCBS in wheat.
The black dots refer to Ka values, the red dots refer to Ks values, and Ka/Ks values are shown by green inverted triangles. The horizontal line and vertical line indicate paralog pairs and the values of Ka, Ks, Ka/Ks, respectively.
